# Voice-controlled autonomous navigation for smart wheelchairs using ROS-based SLAM

**DOI:** 10.1038/s41598-025-34814-6

**Published:** 2026-01-13

**Authors:** Walid Benayed, Mohamed Slim Masmoudi

**Affiliations:** Laboratory SI2E, National Engineering School of Sfax (ENIS), Sfax, 3038 Tunisia

**Keywords:** Engineering, Mathematics and computing

## Abstract

Smart wheelchairs have the potential to significantly improve autonomy for individuals with severe motor impairments, yet existing systems often exhibit limited speech robustness, insufficient handling of dynamic environments, and a lack of rigorously validated safety mechanisms. This work presents a fully integrated, voice-controlled smart wheelchair that advances assistive mobility through three main contributions. First, we introduce an inclusive speech-recognition module built from a fine-tuned deep learning model trained on a custom dataset that incorporates recordings from users with mild speech impairments. This adaptation improves robustness to non-standard pronunciation and maintains reliable command execution under realistic noise conditions (70–75 dB), achieving a Word Error Rate of 6.7% in quiet environments. Second, rather than proposing a new SLAM method, we develop a system-level navigation framework that optimally integrates 2D LiDAR-based SLAM (GMapping), AMCL localization, and a dual-level voice-command interface within a real-time coordination layer. This includes a quantitatively parameterized safety module featuring adaptive speed modulation and experimentally calibrated emergency-stop thresholds, ensuring reliable operation in dynamic indoor environments. Third, we conduct an extensive experimental campaign in both simulation and real-world conditions to provide a reproducible and quantitative evaluation of system performance. Tests involving dynamic obstacles (pedestrians, wheeled carts, small animals), constrained passages, and diverse acoustic settings demonstrate a mean localization error below 10 cm, a 94% goal-completion rate, and an end-to-end voice-to-motion latency of 0.8 s. Together, these contributions provide a low-cost, experimentally validated assistive-mobility platform that emphasizes inclusive voice interaction, robust real-time navigation, and safety-aware behavior. The proposed framework moves beyond component-level studies by offering a coherent, deployable, and reproducible solution for everyday indoor environments.

## Introduction

Wheelchairs have long been an indispensable aid for individuals with motor disabilities. The evolution of models, from manual to powered wheelchairs, is embedded in a history marked by both technological and social advances^1,2^. Historical studies show that these devices are not only tools for mobility but also symbols of integration or exclusion depending on the context^3^. As early as the 1990s, the first studies on intelligent control applied to wheelchairs paved the way for automated and adaptive control solutions^4^.

The history of the powered wheelchair thus illustrates a progressive evolution of mobility aids, from rudimentary models to today’s intelligent devices. On the sociocultural level, Wolfson^3^ analyzes two centuries of wheelchair design, demonstrating that these devices are not merely mechanical tools but also identity and social markers. Furthermore, Cooper’s work^4^ introduced at an early stage the concepts of automated assistance and sensor integration to improve safety and maneuverability. These foundations have served as the basis for contemporary research on smart wheelchairs.

The emergence of these smart wheelchairs addresses the need to go beyond simple motorization by offering intelligent solutions capable of actively assisting the user in their movements. The work of Desai et al.^5^ presents an overview of technological advances in this field, emphasizing the combination of mobile robotics, intelligent sensors, and decision-support algorithms. An important line of research concerns the integration of physiological and brain signals. For example, Ferracuti et al.^6^ propose enhancing wheelchair intelligence through EEG signals, allowing trajectory errors to be avoided by adapting navigation to the user’s intentions. Similarly, Tomari et al.^7^ developed a smart wheelchair system specifically designed for users with severe motor limitations, demonstrating that the design must account for the functional profile and tolerance to error. More recently, several studies have reinforced these directions: Hou et al.^8^ introduced an autonomous wheelchair with integrated IoT-based health monitoring, while Zhewen et al.^9^ explored advanced mobility solutions using Mecanum wheels. Liu et al.^10^ proposed a joystick angle measurement system to better capture user input during powered wheelchair maneuvering. In parallel, brain–computer interface research has progressed toward adaptive control, with studies demonstrating mental state–aware navigation^11^ and hippocampal signal decoding for intention-based guidance^12^. Finally, Hagengruber et al.^13^ highlighted the broader role of assistive robotics in supporting individuals with neurodegenerative conditions, further expanding the scope of intelligent wheelchair research.

Approaches focused on autonomous navigation form another major field. In this context, Bakirci^14^ presented a simulation-based study on autonomous driving for a line-following robotic vehicle, demonstrating how trajectory tracking, sensing, and control strategies can be validated in a realistic simulated environment before deployment. Although not directly related to wheelchairs, this work provides valuable insights into the design and evaluation of autonomous mobility platforms, especially regarding robust trajectory following and sensor integration. Ryu et al.^15^ propose a wheelchair capable of autonomous driving for physically weakened individuals, relying on environmental perception and trajectory planning. Alkhalid and Oleiwi^16^, for their part, highlight the use of voice commands coupled with a tracking system, illustrating the trend toward integrating more natural and accessible interfaces. At the level of reviews, the comprehensive survey by Leaman and La^17^ traces the evolution of intelligent wheelchairs, highlighting technical and ergonomic challenges while opening perspectives for the future. Similarly, Ghorbel et al.^18^ provide a state-of-the-art review of human–machine interactions applied to wheelchairs, revealing the diversity of approaches and underlining the limitations of current solutions.

In parallel with traditional approaches based on manual or voice control, researchers are increasingly exploring advanced interfaces that allow for more natural interaction adapted to the user’s abilities. Among these approaches, gesture control has shown strong potential. Sadi et al.^19^ developed a wheelchair controlled by finger movements, integrated into an IoT platform, demonstrating the feasibility of lightweight and accessible solutions for users with partial hand mobility. Another promising modality is eye-tracking control. Wastlund et al.^20^ proposed a gaze-assisted wheelchair combined with navigation support, offering an effective solution for people unable to use their upper limbs.

The integration of multiple modalities simultaneously, or multimodal interfaces, represents a major step toward truly intelligent wheelchairs. Mahmud et al.^21^ designed an interface combining different control modes (voice, gestures, physiological sensors), demonstrating the effectiveness of hybrid approaches in adapting to users’ specific needs. Moreover, Zhang et al.^22^ studied interactions in crowded environments, developing models enabling intelligent wheelchairs to navigate within groups while accounting for human behavior. These studies highlight that the future of intelligent wheelchairs lies in flexible, customizable, and multimodal interfaces capable of leveraging the complementarity of multiple channels (voice, gestures, gaze, physiological signals).

Autonomous navigation constitutes a fundamental pillar of modern intelligent wheelchairs. It relies on the integration of advanced sensors such as LiDAR (Light Detection and Ranging) and cameras to detect obstacles, estimate position, and plan safe and efficient trajectories^23,24^. Several studies have demonstrated the effectiveness of these sensors in providing smooth navigation in both indoor and urban environments, taking into account user comfort and safety^25,26^. Recent developments further push the boundary of LiDAR-based navigation by integrating deep reinforcement learning for exploration in unknown environments^27^, improving the robustness of LiDAR–inertial–wheel odometry through dynamic point elimination^28^, and introducing more efficient visibility-graph-based path planners for real-time exploration^29^.

The implementation of such navigation is greatly facilitated by the use of ROS (Robot Operating System), which provides a modular and extensible software architecture. ROS manages communication between the system’s various nodes (sensors, perception algorithms, control modules) and leverages existing libraries such as the navigation stack (move_base, amcl, tf, etc.)^30^. Furthermore, the use of SLAM (Simultaneous Localization and Mapping) has become indispensable for enabling the wheelchair to build a map of its environment while localizing itself precisely. Recent research has compared different SLAM algorithms applied to autonomous wheelchairs^31–33^, confirming their importance for operating in unknown and dynamic environments. These approaches pave the way for systems capable of adapting to varied contexts, from confined spaces^32^ to complex urban environments^23^.

Although the literature demonstrates significant progress in the field of intelligent wheelchairs, several challenges remain. On the one hand, existing voice control systems still show limitations in terms of robustness and reliability in noisy environments^16,17^. On the other hand, autonomous navigation solutions, while effective in controlled contexts, require deeper integration with modular software platforms such as ROS in order to ensure adaptability to dynamic and cluttered environments^23,24,30^. Finally, multimodal approaches^19–22^ open promising perspectives, but they remain complex to implement and difficult to generalize to all user profiles.

In this context, our article proposes an innovative approach that combines a deep learning–based voice command with autonomous navigation integrated into ROS and reinforced by the use of SLAM. More specifically, our contributions are structured into four main components:

- Design of a robust voice recognition module, based on a deep learning model (VOSK), capable of understanding simple commands adapted to the user’s abilities.
- Software integration with ROS, enabling smooth communication between the voice command module, the sensors (notably a LiDAR), and the navigation stack, to ensure a modular and scalable architecture.
- Implementation of SLAM (Simultaneous Localization and Mapping) algorithms for real-time mapping and localization. This approach allows the wheelchair to operate in unknown environments, avoid obstacles, and generate a dynamic map, consistent with recent advances^25,26,31–33^.
- Experimental validation in simulation and real-world environments, including autonomous navigation tests in a structured space (ENIS nursery), with performance evaluation in terms of accuracy, recognition rate, response time, and safety.

This approach stands out for its aim to develop an accessible, autonomous, and intelligent system, capable of significantly improving user experience by enhancing autonomy and social inclusion. Thus, our work builds upon existing research^5,15,17,23^, while proposing an integrated solution that coherently combines voice control, ROS-based autonomous navigation, and SLAM mapping in a practical way.

## Related work

### Evolution of smart wheelchairs

The evolution of wheelchairs has followed a progressive trajectory, from rudimentary manual models to powered wheelchairs, and more recently to current intelligent solutions. In the early 1990s, the pioneering work of Cooper^4^ on intelligent control paved the way for the integration of sensors and decision-support algorithms. Subsequently, several historical studies^1,3^ have shown that the wheelchair is not only a tool for mobility but also a symbol of autonomy and social integration.

With the emergence of *smart wheelchairs*, research has focused on the integration of perception technologies (LiDAR, cameras, physiological sensors), control methods (adaptive joysticks, voice commands), and autonomous navigation. Desai et al.^5^ summarized these advances, while Leaman and La^17^ proposed a comprehensive review tracing the evolution of solutions and identifying remaining challenges. More recently, attention has been directed toward multimodal approaches, combining multiple interfaces such as voice, gesture, and gaze to enable more natural interaction^19–22^.

### Existing projects and solutions (VAHM, WAD, etc.)

Numerous research projects and prototypes have been proposed to improve wheelchair accessibility and autonomy. Among them, the *VAHM (Voice Activated Head Motion)* and *WAD (Wheelchair Autonomous Driving)* systems stand out for their innovative approaches. VAHM combines voice recognition with head movements to control a wheelchair, making it suitable for users with limited upper-limb mobility. WAD, on the other hand, emphasizes autonomous navigation powered by ROS and enhanced with perception and mapping algorithms, enabling real-time obstacle avoidance.

Recent work confirms these trends: the *CoNav Chair (2025)* project introduces shared control based on ROS and 3D SLAM, combining safety and comfort for the user^34^. The study by Cui et al. (2024) proposes multimodal interaction (voice, gestures, head posture) coupled with autonomous navigation using LiDAR and SLAM, demonstrating high positioning accuracy^35^. Other innovative approaches include the integration of artificial intelligence for environmental recognition and adaptive trajectory planning, such as systems combining SLAM with YOLO (You Only Look Once)for dynamic obstacle avoidance^36^.

### Identified limitations in the literature

Despite significant advances, several limitations remain in the design and implementation of smart wheelchairs:

- *Robustness of voice commands* speech recognition systems remain sensitive to background noise as well as variations in accent or pronunciation^16,17^.
- *Complexity of multimodal interfaces* while gesture-, gaze-, or EEG-based solutions offer greater flexibility, their implementation remains complex and difficult to generalize^19–22^.
- *Autonomous navigation in dynamic environments* although ROS and SLAM have improved planning and localization, challenges remain in crowded or urban spaces, particularly regarding real-time adaptation^23–26,31–33^.
- *Lack of large-scale user evaluations* several projects remain limited to prototypes tested in simulation or laboratory conditions, without thorough validation involving end-users^17^.

These limitations justify the need for integrated solutions that combine robustness, flexibility, and adaptability. In this context, our work aims to strengthen the reliability of voice control through deep learning, while leveraging ROS and SLAM for truly autonomous navigation adapted to real-world environments.

## Methodology

### General system approach

The methodological approach proposed in this work is based on the design of a smart wheelchair system that integrates both an artificial intelligence-based voice command interface and autonomous navigation relying on the ROS framework and SLAM algorithms. The guiding principle is to provide the user with a mobility tool that combines ease of use, autonomous movement, and enhanced safety in complex environments, while remaining accessible to individuals with severe motor impairments.

The system is characterized by an integrated functional chain that begins with capturing the user’s intention through voice. This command is processed by a speech recognition module built on deep learning models capable of recognizing a limited but domain-specific driving vocabulary. This stage is particularly critical in noisy environments, where robustness and accuracy of recognition determine the overall reliability of the system. Once the command is correctly identified, it is translated into a high-level instruction and transmitted to the central manager running under ROS.

ROS (Robot Operating System) serves as the software backbone of the architecture. It not only ensures communication between the different system nodes (voice command, perception, planning, and action), but also provides proven libraries for mobile navigation. The onboard sensors, particularly the LiDAR rangefinder, continuously transmit data about the wheelchair’s immediate surroundings. These inputs feed into a SLAM (Simultaneous Localization and Mapping) module responsible for constructing and updating a map of the environment while simultaneously localizing the wheelchair within it. This dual function of mapping and localization is crucial for enabling reliable autonomous movement, even in unknown or dynamic environments.

The ROS navigation stack then exploits this information to generate safe and optimal trajectories. It combines global planning, which aims to find the best path toward the goal implicitly defined by the user’s voice command, with local planning, which continuously adapts movement based on real-time obstacle detection. This hierarchical approach ensures both efficiency (optimized paths) and safety (dynamic obstacle avoidance).

The execution of the calculated trajectories is handled by a low-level control layer that drives the wheelchair’s actuators. This loop operates in real time, guaranteeing a smooth and natural response to commands. The user is thus freed from the need for continuous intervention: a simple instruction such as “move forward,” “turn left,” or “go to the kitchen” is sufficient to trigger a complete sequence of perception, planning, and autonomous navigation.

**Fig. 1:**
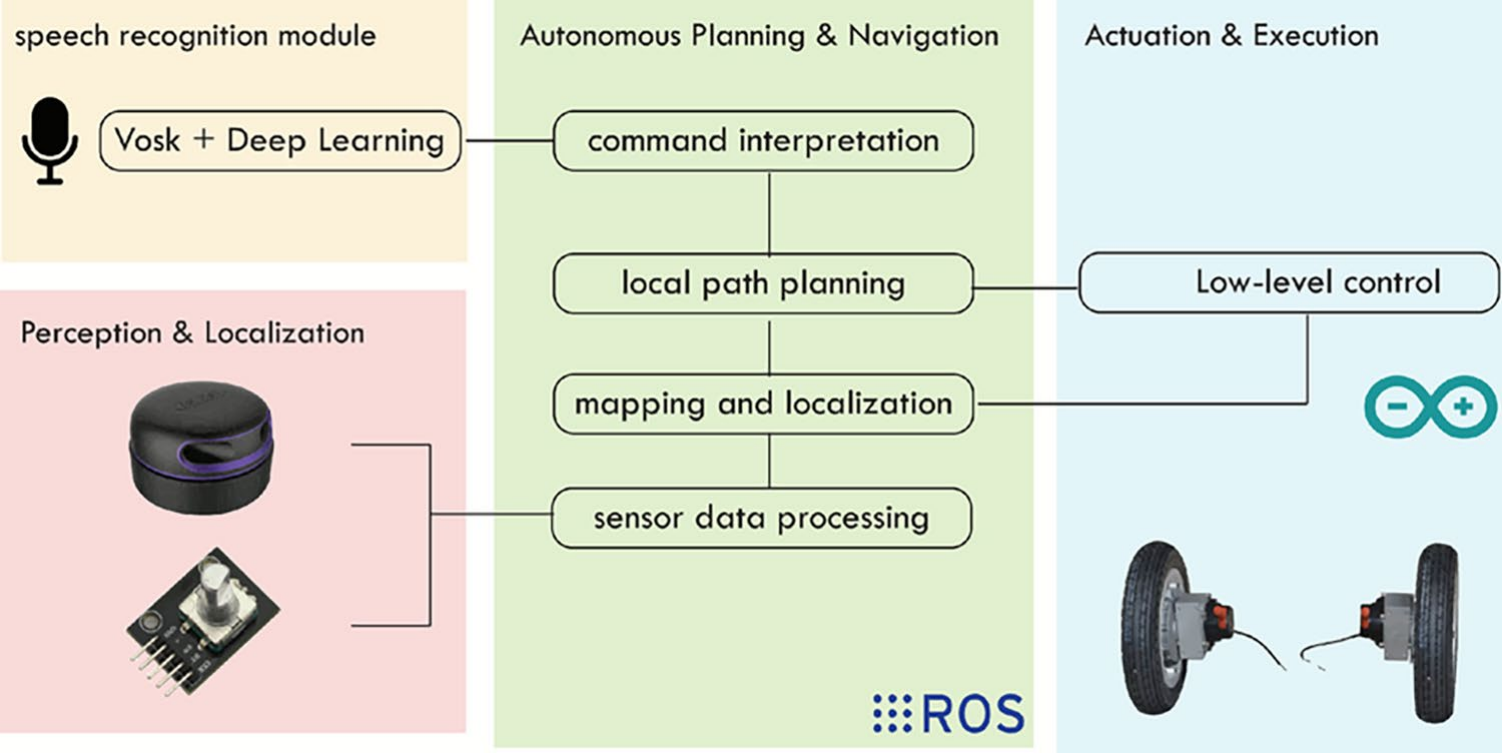
Overall system architecture of the intelligent wheelchair.

In summary, the general approach is founded on the harmonious integration of an intuitive communication interface (voice command), a powerful and modular software architecture (ROS), and advanced perception and localization algorithms (SLAM). Together, these elements form a coherent, scalable, and extensible system capable of addressing diverse user needs, while also paving the way for future extensions such as the inclusion of multimodal interfaces or the integration of shared-control modules. The overall architecture of the proposed system is illustrated in Fig. 1.

### Hardware setup

The hardware architecture of the smart wheelchair was designed to ensure a balance between computational power, perception accuracy, and energy efficiency. Each component was selected for its technical relevance and suitability for the requirements of autonomous navigation and real-time voice interaction.

*Processing Unit* The NVIDIA Jetson Nano serves as the main processing unit. It is equipped with a quad-core ARM Cortex-A57 CPU, a Maxwell GPU with 128 CUDA cores, and 4 GB of LPDDR4 memory. Its modest power consumption (5–10 W) allows integration into a mobile system, while still providing sufficient computing power for deep learning algorithms and navigation under ROS. Its major advantage lies in its compatibility with AI libraries (TensorFlow, PyTorch, OpenCV) as well as with the ROS ecosystem.

Low-level Control Microcontroller: For motor and odometry sensor management, an Arduino UNO, based on the ATmega328P microcontroller (16 MHz, 32 KB flash memory), was chosen. It ensures low-level control and relieves the Jetson Nano from real-time tasks. Its ease of use and wide adoption in the scientific community make it a robust and easily reproducible choice.

*Perception and odometry sensors* Environmental perception is provided by a YDLIDAR X4PRO 2D LiDAR, capable of performing $360^{\circ }$ measurements with a range of up to 12 m and a scanning frequency between 5 and 12 Hz. It represents an ideal compromise between cost and performance, particularly for SLAM applications. In addition, a *KY-040 rotary encoder* is used for odometry, providing estimates of wheel speed and relative position, thereby improving localization when fused with LiDAR data.

*Voice Interface* A directional USB microphone is integrated to capture voice commands. This sensor ensures reliable acquisition and reduces off-axis noise interference, while being easily connectable to the Jetson Nano thanks to its Plug-and-Play USB interface.

Table 1 summarizes the main components used, their technical specifications, and their advantages.

**Table 1:** Summary of hardware components of the proposed system.

Component	Technical specifications	Main advantages
NVIDIA Jetson Nano	ARM Cortex-A57 Quad-core CPU; Maxwell GPU 128 CUDA cores; 4 GB LPDDR4 RAM; Power 5–10 W	Real-time AI processing; ROS and AI libraries compatibility; Low power consumption
Arduino UNO (ATmega328P)	16 MHz frequency; 32 KB flash memory; 14 digital I/O pins	Reliable low-level control; Large community support; Easy integration
LiDAR YDLIDAR X4PRO	Max. range 12 m; $360^{\circ }$ coverage; 5–12 Hz scan frequency; Millimeter precision	Excellent cost/performance ratio; SLAM compatibility; Reliable indoor navigation
Directional USB Microphone	USB Plug-and-Play interface; Axial directivity	Clear voice capture; Noise reduction; Direct integration with Jetson Nano

This hierarchical hardware organization, based on a clear separation between high-level processing and low-level control, ensures system robustness and efficiency while facilitating scalability for the integration of future sensors.

### Deep learning-based voice command

The proposed voice command module was specifically designed to meet the requirements of real-time embedded control of a smart wheelchair. Unlike generic speech recognition systems, our objective was not to cover a large vocabulary, but to optimize recognition for a small set of navigation commands while ensuring robustness in noisy indoor environments.

To this end, we implemented a deep learning-based pipeline running fully offline on the Jetson Nano. The design choices were motivated by three practical constraints of the application:

- *Low latency* commands must be recognized and executed within less than one second to guarantee smooth interaction.
- *Noise robustness* the system must remain operational in environments with background noise (corridors, laboratories, incubator testbeds).
- *Resource efficiency* the model must be compact enough to run in real time on an embedded platform without cloud support.

The resulting implementation relies on the Vosk engine as a lightweight inference framework, extended with a fine-tuned deep neural network adapted to the wheelchair use case. The network was trained and evaluated on a custom dataset including multiple speakers, accents, and varying acoustic conditions. This specialization allowed us to significantly reduce the Word Error Rate (WER) compared to the generic pretrained model, while maintaining compatibility with the limited computational resources of the embedded hardware.

#### Architecture of the recognition model

The proposed speech recognition system was implemented using the *Vosk* library, an open-source engine optimized for embedded platforms such as the Jetson Nano. The processing pipeline begins with audio acquisition through a directional microphone. The captured signal is first preprocessed to reduce environmental noise and segmented into short overlapping frames. For each frame, Mel-Frequency Cepstral Coefficients (MFCC) are extracted, providing a compact representation of the spectral envelope of speech, which is widely adopted in state-of-the-art recognition systems.

The extracted features are then processed by a deep neural network composed of convolutional and recurrent layers. The convolutional layers act as local feature extractors, identifying robust spectral patterns while ensuring invariance to small shifts in frequency and time. The output of the convolutional stage is passed to recurrent layers, based on Long Short-Term Memory (LSTM) or Gated Recurrent Unit (GRU) cells, which capture the temporal dependencies of speech and encode the sequential nature of the signal. This hierarchical architecture therefore combines local spectral analysis with long-range temporal modeling, which is essential for reliable command recognition.

At the output, the network produces a sequence of posterior probabilities over a predefined vocabulary, including characters and a special blank token. Decoding is performed using the Connectionist Temporal Classification (CTC) framework, which aligns the variable-length input sequences with the corresponding target transcriptions. The CTC loss is defined as:

1 $$\begin{aligned} \mathscr {L}_{CTC}(x, y) = - \ln \sum _{\pi \in \mathscr {B}^{-1}(y)} p(\pi \mid x), \end{aligned}$$

where *x* denotes the input sequence of acoustic features, *y* is the target transcription, $\pi$ represents a possible alignment path, and $\mathscr {B}$ is the mapping function that removes blanks and repeated symbols. This formulation makes it possible to train the model without the need for explicit frame-level annotations, which are costly to obtain.

**Fig. 2:**
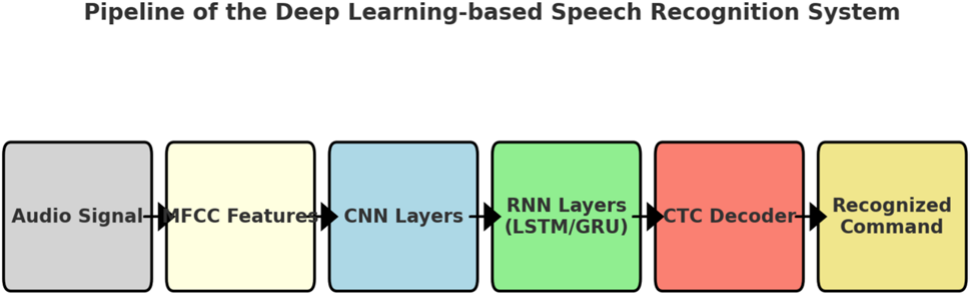
Pipeline of the proposed speech recognition module: the audio signal is converted into MFCC features, processed through CNN and RNN layers, and decoded by the CTC framework into textual commands.

This CNN–RNN–CTC architecture, inspired by successful implementations such as DeepSpeech and Kaldi, constitutes the current standard for embedded speech recognition. Its main advantage lies in its ability to handle variable-length sequences while remaining compact enough to operate in real time on low-power platforms such as the Jetson Nano. The overall processing pipeline of the proposed speech recognition module is illustrated in Fig. 2.

#### Dataset and preprocessing

The development of a robust speech recognition module requires an appropriate dataset that accurately reflects the acoustic variability encountered in real-world use. To this end, we relied on a combination of generic open-source corpora and a custom dataset specifically collected under representative conditions of wheelchair operation. The custom dataset comprised approximately 1,200 audio samples recorded from ten speakers (six male and four female) with diverse accents and timbres. Each speaker pronounced the defined set of five navigation commands (*“forward”*, *“backward”*, *“left”*, *“right”*, and *“stop”*) in three different environments–corridors, laboratories, and shared spaces–with varying levels of background noise. This design ensured that the training data captured both inter-speaker variability and realistic acoustic disturbances relevant to daily wheelchair usage.

In addition to this core dataset, an auxiliary set of speech samples was collected in collaboration with a rehabilitation center. This supplementary corpus included recordings from four individuals with documented speech impairments (mild dysarthria or atypical articulation). All recordings were anonymized according to institutional guidelines. These samples were used to expose the model to non-standard pronunciation patterns typically encountered among users with motor impairments, thereby enhancing the inclusiveness and robustness of the system.

Before being fed into the neural network, the raw audio waveforms underwent a series of preprocessing steps. The signals were first segmented into short overlapping frames of 25 ms with a 10 ms stride, preserving the quasi-stationary properties of speech. Each frame was then windowed using a Hamming filter to minimize spectral leakage.

For feature extraction, Mel-Frequency Cepstral Coefficients (MFCC) were computed to obtain a perceptually motivated representation of the speech spectrum. Each frame was represented by a 13-dimensional MFCC vector, augmented with first- and second-order derivatives to capture temporal dynamics, yielding a 39-dimensional feature vector. These features were subsequently normalized using mean and variance normalization to ensure robustness to amplitude variations across speakers and recording sessions.

To further enhance robustness—particularly under noisy conditions—data augmentation techniques were applied. Artificial background noise at different signal-to-noise ratios (SNRs) was added to a subset of the training corpus, while pitch shifting was used to simulate variations in vocal timbre. These augmentations improved the model’s generalization capacity, enabling stable recognition performance in acoustically diverse environments.

This preprocessing pipeline yielded compact, normalized, and noise-robust feature representations that served as the input to the deep neural network described in the previous section.

#### Training and fine-tuning strategy

While the Vosk engine provides pretrained models trained on large-scale multilingual corpora, their direct application to the smart wheelchair scenario proved suboptimal. In particular, the limited vocabulary of navigation commands and the presence of background noise in the experimental environment required a domain-specific adaptation. In this work, the auxiliary impaired-speech recordings collected at Hédi Chaker Hospital were also incorporated into the fine-tuning stage to better account for atypical pronunciation patterns and improve robustness for users with speech impairments.

To address these challenges, a fine-tuning phase was carried out on the collected dataset described previously. The pretrained acoustic model was initialized with existing parameters and updated using a small learning rate of $1 \times 10^{-4}$ to avoid catastrophic forgetting. The training process was conducted for 15 epochs with a batch size of 32, and early stopping was applied based on the validation loss to prevent overfitting.

The optimization was performed using the Adam optimizer, with a gradual decay of the learning rate across epochs. During fine-tuning, the lower convolutional layers were kept frozen to preserve their generic feature extraction capabilities, while the higher recurrent layers and the CTC output layer were retrained to adapt to the specific pronunciation and noise characteristics of the target domain, including the atypical articulation patterns present in the hospital data.

Data augmentation, including noise injection and pitch shifting, was maintained during training to maximize robustness. This strategy allowed the model to specialize in the recognition of a reduced vocabulary of commands without losing its generalization ability.

The effectiveness of this fine-tuning procedure was confirmed by a significant reduction in Word Error Rate (WER), which decreased from 12.3% with the generic model to 6.7% with the adapted model. This performance improvement directly translated into smoother user interaction and greater reliability in real-world tests, as described in the performance evaluation section.

Thus, the adopted fine-tuning strategy demonstrates that even lightweight embedded speech recognition engines can achieve near state-of-the-art performance when properly adapted to their application context.

#### Low-level and high-level commands

A distinctive aspect of the proposed speech recognition module lies in the organization of its vocabulary into two complementary levels of control. This design choice reflects the need to reconcile the immediate reactivity of direct control with the ergonomic advantages of autonomous navigation, thereby ensuring that the system remains usable across a wide spectrum of contexts and user profiles.

The first level, referred to as low-level commands, consists of a minimal set of primitive instructions such as *forward*, *backward*, *left*, *right*, and *stop*. These commands are mapped to basic movement actions that are executed instantaneously after recognition. This mode of interaction is comparable to the functionality of a virtual joystick, with the essential difference that it relies entirely on voice rather than on residual muscular control. Such a strategy is particularly relevant in scenarios that demand precise maneuvering or quick corrections, for example when avoiding furniture in a domestic environment or aligning the wheelchair with a narrow doorway. In this sense, low-level commands provide the user with an enhanced sense of agency and fine-grained control over motion, even in complex or cluttered spaces.

The second level introduces high-level commands, which enrich the interaction by allowing the specification of semantic destinations instead of individual movements. In this case, the user can simply pronounce labels such as *kitchen*, *bedroom*, or *training room*. Each label is associated with a predefined location on the occupancy map constructed by the SLAM module. Once such a command is detected, the system autonomously generates a navigation goal and computes an optimal trajectory towards it, while continuously adapting to dynamic obstacles along the way. By abstracting the navigation process, high-level commands considerably reduce the cognitive and physical effort required from the user, particularly for long-distance or repetitive displacements.

The coexistence of these two levels of control illustrates a hybrid approach to human–robot interaction. Rather than imposing a single mode of operation, the system provides users with the flexibility to choose between direct intervention and delegated autonomy, depending on their immediate needs and preferences. From an accessibility perspective, this duality is of paramount importance: individuals with greater residual abilities may prefer the reactivity of low-level control, while those with severe impairments can rely more extensively on high-level navigation commands.

Beyond usability, this dual strategy contributes to safety and robustness. Low-level control ensures that the user can override or fine-tune the behavior of the wheelchair in unforeseen situations, while high-level control leverages the navigation stack to guarantee globally efficient and collision-free trajectories. Taken together, these two complementary modes form a coherent framework that combines autonomy with human-centered flexibility, thereby advancing the state of the art in assistive mobility systems.

#### Performance evaluation

The performance of the speech recognition module was evaluated using both objective and application-oriented criteria. The main metric adopted was the Word Error Rate (WER), which quantifies the percentage of insertions, deletions, and substitutions with respect to the reference transcriptions.

Experiments were first conducted with the generic pretrained model provided by Vosk. While recognition accuracy was satisfactory in quiet conditions, performance degraded significantly in noisy environments, with WER values exceeding 20%. In contrast, the fine-tuned model demonstrated a marked improvement, achieving an average WER of 6.7% in quiet conditions and maintaining acceptable accuracy under moderate noise levels (65 dB).

To place these results in perspective, we also compared the proposed system with two alternative approaches: the generic Vosk model and a cloud-based solution (Google Speech API). Table 2 summarizes the results.

**Table 2:** Comparison of speech recognition models in terms of WER and latency.

Model	WER (Quiet) (%)	WER (Noisy 65 dB) (%)	Latency (s)
Generic Vosk model	12.3	20.8	0.6
Fine-tuned model (ours)	**6.7**	**11.5**	0.8
Google speech API	5.9	10.7	1.2

The results indicate that the fine-tuned embedded model achieves a level of accuracy comparable to cloud-based systems, while maintaining the advantage of operating fully offline. The latency, measured as the delay between the end of a spoken command and its execution, remained below one second in all cases, which is compatible with real-time wheelchair control.

These findings confirm that the proposed system meets the dual objective of robustness and responsiveness, which are essential for user acceptability in assistive applications.

### Autonomous navigation based on ROS and SLAM

#### General principle of autonomous navigation

Autonomous navigation is a central capability of the proposed system, enabling the smart wheelchair to operate safely in indoor environments without continuous manual input. The implementation relies on the *ROS (Robot Operating System)* framework, which provides the modular infrastructure supporting perception, localization, planning, and motion execution. In this section, we focus on the system-specific integration of these components rather than on standard ROS mechanisms.

The navigation pipeline combines well-established ROS packages with modules developed specifically for assistive voice-controlled mobility. The SLAM subsystem employs the GMapping package, which implements a Rao–Blackwellized particle filter^37^. GMapping was selected after evaluating alternatives such as Cartographer, HectorSLAM, and RTAB-Map. While these approaches offer strong performance in large-scale or multimodal scenarios, they require significantly higher computational resources or exhibit increased sensitivity to noise. In contrast, GMapping provides a robust compromise between accuracy and real-time capability on embedded platforms such as the NVIDIA Jetson, producing stable 2D occupancy-grid maps and reliable loop closure for low-speed indoor navigation.

Localization is performed using Adaptive Monte Carlo Localization (AMCL), based on the KLD-sampling method^38^. Global planning relies on Dijkstra or A*, while local planning uses the Dynamic Window Approach (DWA)^39^. These standard components operate within the broader ROS architecture^40^, with configurations adapted to the wheelchair’s kinematics and sensing profile.

Beyond these standard modules, several elements were developed specifically for this system. A fully integrated voice-command interface based on the Vosk ASR engine interprets user instructions in real time and translates them into navigation goals. A dynamic-object filtering mechanism based on temporal scan consistency increases SLAM robustness in environments containing moving pedestrians. Furthermore, a coordination layer synchronizes SLAM, AMCL, global and local planning, obstacle avoidance, and motor control to ensure coherent behavior and fault tolerance.

During operation, onboard sensors—primarily LiDAR, complemented by optional depth sensing—acquire environmental data that feed the SLAM module to simultaneously update the map and estimate the wheelchair’s pose. This capability enables operation in previously unknown environments and continuous refinement of localization in mapped areas.

To verify suitability for assistive navigation, we assessed the consistency of the SLAM subsystem across multiple mapping trials. The generated occupancy grids showed low structural deviation (5–7 cm), loop-closure events were consistently triggered, and CPU usage remained within 18–25%, confirming stable performance under embedded real-time constraints.

Once localization is established, the navigation stack computes safe and feasible trajectories. The global planner identifies an optimal path to the user-defined goal, while the local planner adapts commands to avoid static or dynamic obstacles. Motion commands are then translated into low-level motor inputs, closing the perception–planning–action loop. This architecture enables the wheelchair to continuously analyze its surroundings and autonomously execute the destination specified through voice commands.

#### Integration of ROS and the navigation stack

The integration of ROS constitutes the backbone of our autonomous navigation system by coordinating perception, localization, planning, and control modules within a unified operational framework. Rather than describing standard ROS mechanisms, we focus here on the system-specific configuration and interaction of these components as implemented in the smart wheelchair.

The ROS navigation stack merges three primary inputs: the real-time occupancy grid produced by the SLAM module, the pose estimate generated by the AMCL localization unit, and the navigation goal interpreted from voice commands and forwarded to the /move_base interface. This fusion of mapping, probabilistic localization, and high-level intent forms the basis of the perception–planning pipeline.

To ensure reproducibility, the AMCL module was configured with 600 particles, providing a trade-off between localization accuracy and embedded computational constraints. The motion model parameters $(\alpha _1 = 0.02,\ \alpha _2 = 0.02,\ \alpha _3 = 0.01,\ \alpha _4 = 0.01)$ account for rotational and translational noise, while the beam_model uses a Gaussian hit component with $\sigma _{\text {hit}} = 0.03$ m and a random measurement probability of $z_{\text {rand}} = 0.15$. Adaptive resampling was enabled with an effective particle ratio of 0.5. AMCL operated at 10 Hz on a SLAM-generated map with 0.05 m resolution. These parameters define the localization behavior and provide the methodological clarity requested by the reviewer.

Given the pose estimate and map, the global planner computes a collision-free path using graph-search methods such as Dijkstra or A*. The Dynamic Window Approach (DWA) local planner then refines the trajectory by evaluating dynamically feasible velocity commands and selecting those that optimize a multi-objective cost function balancing obstacle clearance, target alignment, and smoothness of motion. This enables real-time adaptation to dynamic obstacles detected by the LiDAR.

The resulting linear and angular velocity commands $(v, \omega )$ are transmitted to the motor controller, closing the perception–planning–action loop. As sensor data are continuously updated, SLAM refines the map and AMCL adjusts the pose estimate, allowing the navigation stack to re-evaluate and update the trajectory when environmental changes occur.

This hierarchical and modular integration ensures that high-level voice instructions are reliably translated into safe, reactive, and fully autonomous navigation behaviors, while remaining compatible with the computational limits and safety requirements of assistive mobility.

#### Use of SLAM for mapping and localization

The wheelchair’s autonomous navigation relies on the use of *SLAM (Simultaneous Localization and Mapping)* algorithms, whose objective is to build a map of the environment while estimating in real time the robot’s position within it. This capability is fundamental, as it enables the system to operate in both unknown environments and partially mapped spaces.

In our architecture, the SLAM module receives as input data from the LiDAR sensor, optionally complemented by information from a depth camera . These sensors provide a rich representation of the immediate environment, enabling the detection of both static obstacles and surface variations. The SLAM algorithm uses these data to generate an incremental map, represented as an occupancy grid, that describes free areas, obstacles, and unknown regions.

In parallel with mapping, the module continuously estimates the wheelchair’s position using probabilistic localization. In our case, the approach relies on AMCL (*Adaptive Monte Carlo Localization*), which uses a particle filter to model positional uncertainty and correct it as new observations become available. This method ensures robust localization, even in the presence of sensor noise or ambiguities in symmetric environments.

The combination of mapping and localization allows the ROS *navigation stack* to generate optimal trajectories to the user’s destination. The SLAM-generated map serves as the basis for the global planner to compute the path, while localization ensures that the wheelchair remains aligned with this path throughout the journey.

Thus, the use of SLAM is a crucial component of our methodology: it ensures that the wheelchair does not operate blindly but always has a coherent and up-to-date representation of its environment. This functionality also opens the way to future extensions, such as navigation in dynamic environments, the integration of visual SLAM for richer perception, or multisensor fusion to further improve the system’s accuracy and resilience.

#### Safety and obstacle avoidance

User safety is a fundamental requirement in the design of a smart wheelchair. For this reason, the proposed architecture integrates an obstacle avoidance module capable of continuously adapting planned trajectories according to environmental conditions. This functionality relies on onboard sensors, primarily the LiDAR, which provides a detailed real-time representation of the surrounding space in the form of a point cloud. These data feed into the local costmap, which is used by the planner of the ROS *navigation stack*.

The avoidance mechanism operates hierarchically. First, the global planner defines an optimal path on the map generated by SLAM. Then, the local planner, such as the Dynamic Window Approach (DWA) algorithm, dynamically adjusts the trajectory to prevent collisions with static or moving obstacles. This approach reconciles two complementary objectives: adherence to the initial path and the ability to adapt to unforeseen circumstances.

To further enhance robustness, a critical detection logic is integrated. When an obstacle appears at a distance below a safety threshold, the wheelchair automatically slows down and, if necessary, comes to a complete stop. This rapid reaction ensures that even in the case of an incorrect voice instruction or a temporarily disturbed sensor, user safety is never compromised.

In practice, experiments have shown that this reactive architecture enables smooth movement while significantly reducing the risk of collision. The user thus benefits from intuitive control through voice commands, while the autonomous layer guarantees a high level of safety and reliability in real environments. This combination of human interaction and artificial intelligence is a key factor for the acceptability and trust in the daily use of the smart wheelchair.

### Environment perception

Environmental perception plays a crucial role in the autonomous navigation of a smart wheelchair. It not only enables the detection of obstacles in the surrounding space but also provides accurate information on the system’s position and motion. In our architecture, perception relies primarily on the integration of a LiDAR sensor and odometry data, fused within the ROS navigation framework.

#### Odometry modeling

The wheelchair’s odometry is derived from incremental rotary encoders mounted on the two driving wheels. Let *r* denote the wheel radius, *d* the wheelbase (distance between wheels), and $\Delta \phi _R$, $\Delta \phi _L$ the incremental angular displacements of the right and left wheels, respectively.

The linear displacement $\Delta s$ and the angular rotation $\Delta \theta$ are expressed as:

2 $$\begin{aligned} \Delta s= & \frac{r}{2} \left( \Delta \phi _R + \Delta \phi _L \right) , \end{aligned}$$

3 $$\begin{aligned} \Delta \theta= & \frac{r}{d} \left( \Delta \phi _R - \Delta \phi _L \right) . \end{aligned}$$

Considering the current pose $(x_k, y_k, \theta _k)$, the updated pose $(x_{k+1}, y_{k+1}, \theta _{k+1})$ is given by:

4 $$\begin{aligned} x_{k+1}= & x_k + \Delta s \cdot \cos \!\left( \theta _k + \tfrac{\Delta \theta }{2}\right) , \end{aligned}$$

5 $$\begin{aligned} y_{k+1}= & y_k + \Delta s \cdot \sin \!\left( \theta _k + \tfrac{\Delta \theta }{2}\right) , \end{aligned}$$

6 $$\begin{aligned} \theta _{k+1}= & \theta _k + \Delta \theta . \end{aligned}$$

**Fig. 3:**
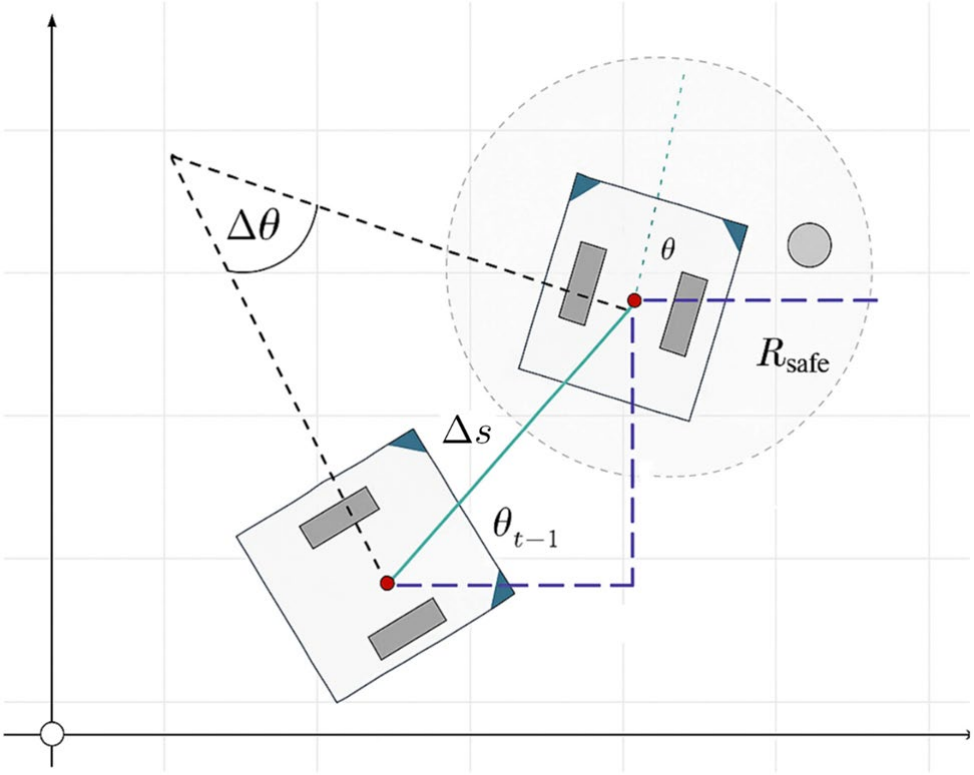
Illustration of the wheelchair kinematic model: transition between two successive poses $(x, y, \theta )$, orientation change $\Delta \theta$, displacement *d*, and consideration of the safety radius $R_{safe}$.

The kinematic model of the wheelchair motion is illustrated in Fig. 3. The figure shows the displacement *d* between two consecutive poses, the orientation change $\Delta \theta$, and the updated heading $\theta$. In addition, a safety radius $R_{safe}$ is considered around the wheelchair to ensure collision-free navigation in dynamic environments.

**Fig. 4:**
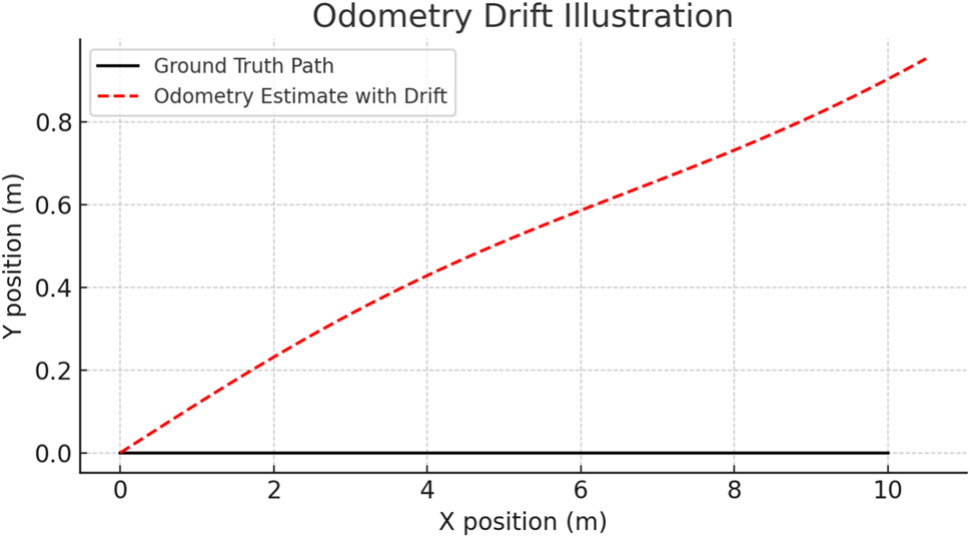
Illustration of odometry drift: deviation between estimated trajectory and ground truth path over time.

This kinematic model provides a real-time estimate of the wheelchair trajectory based on wheel motion. However, odometry errors accumulate over time due to wheel slip, uneven surfaces, and encoder noise. This phenomenon is illustrated in Fig. 4, where the estimated trajectory gradually deviates from the ground truth path.

Therefore, odometry alone is insufficient for long-term navigation, motivating its fusion with LiDAR and SLAM modules to maintain global consistency.

#### Localization with SLAM and AMCL

Odometry alone provides only short-term accuracy, as it is subject to cumulative drift. To overcome this limitation, odometry is fused with LiDAR data within the ROS framework using the *Simultaneous Localization and Mapping* (SLAM) module for map generation, and the *Adaptive Monte Carlo Localization* (AMCL) algorithm for pose estimation.

AMCL implements a particle filter that estimates the posterior belief of the robot’s pose $x_t$ given all past controls $u_{1:t}$ and observations $z_{1:t}$:

7 $$\begin{aligned} bel(x_t) = p(x_t \mid z_{1:t}, u_{1:t}) . \end{aligned}$$

This belief is recursively updated in two steps:

*Prediction (motion model):*

8 $$\begin{aligned} \tilde{bel}(x_t) = \int p(x_t \mid u_t, x_{t-1}) \, bel(x_{t-1}) \, dx_{t-1}, \end{aligned}$$

where $p(x_t \mid u_t, x_{t-1})$ models the uncertainty of odometry.

*Correction (measurement update):*

9 $$\begin{aligned} bel(x_t) = \eta \, p(z_t \mid x_t, m) \, \tilde{bel}(x_t), \end{aligned}$$

where $p(z_t \mid x_t, m)$ measures the likelihood of the LiDAR scan $z_t$ given the map *m*, and $\eta$ is a normalization constant.

**Fig. 5:**
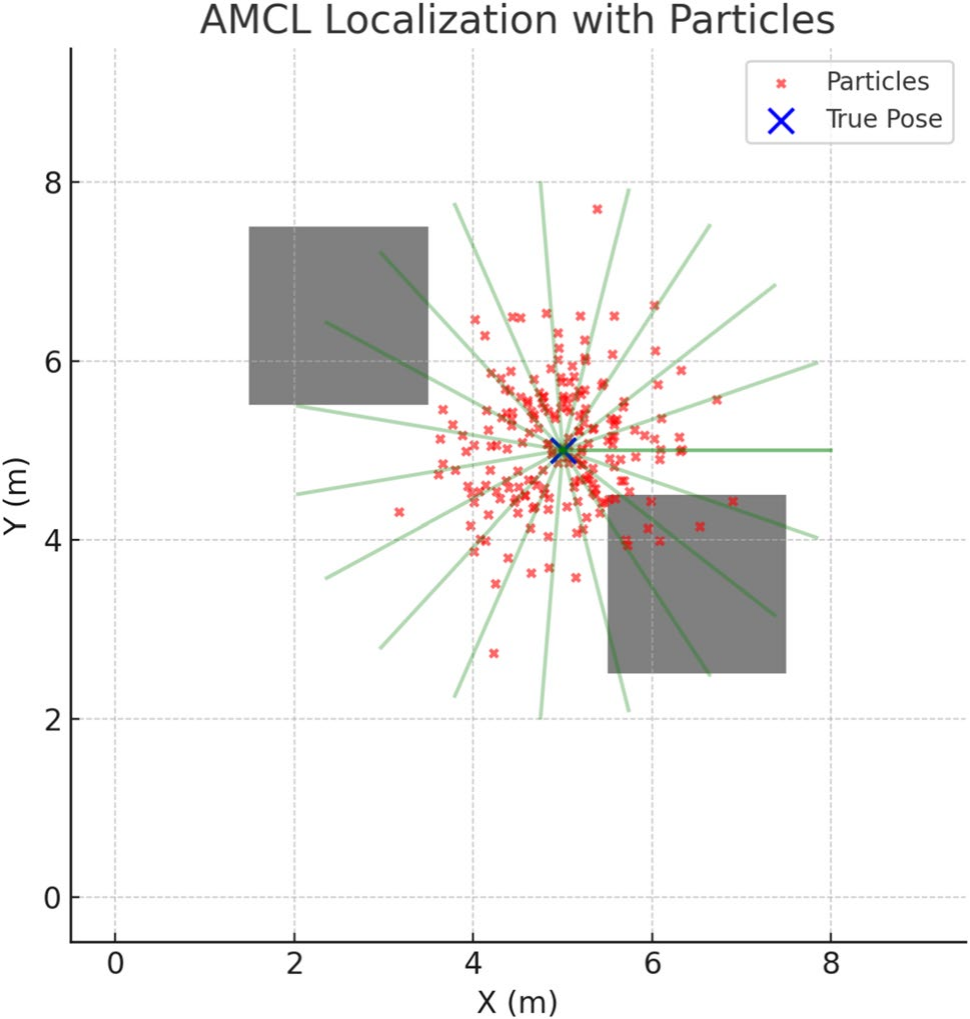
Illustration of AMCL: LiDAR scan alignment with the occupancy grid, with particles representing pose hypotheses.

Through resampling, particles with higher likelihood survive, while improbable poses are eliminated. This allows the wheelchair to remain localized even in complex or symmetric environments, as illustrated in Fig. 5.

This probabilistic fusion of odometry and LiDAR guarantees that the wheelchair maintains a consistent and accurate pose estimate, which is essential for trajectory planning and obstacle avoidance.

#### Obstacle detection and safety mechanism

The main perception sensor is the 2D YDLIDAR X4PRO, which performs a $360^{\circ }$ scan with a maximum range of 10 m and a frequency between 5 and 12 Hz. Each scan provides polar coordinates $(r_i, \theta _i)$ that are transformed into Cartesian coordinates as:

10 $$\begin{aligned} x_i = r_i \cdot \cos (\theta _i), \quad y_i = r_i \cdot \sin (\theta _i). \end{aligned}$$

The resulting point cloud is integrated into the ROS *navigation stack* as local costmaps. For each detected obstacle, the Euclidean distance to the wheelchair’s center is computed as:

11 $$\begin{aligned} d_i = \sqrt{(x_i - x_r)^2 + (y_i - y_r)^2}, \end{aligned}$$

where $(x_r, y_r)$ is the wheelchair’s current position.

A dynamic safety radius $R_{safe}$ is defined according to the wheelchair’s velocity *v*:

12 $$\begin{aligned} R_{safe}(v) = R_0 + k \cdot v, \end{aligned}$$

where $R_0$ is the minimum static clearance and *k* is a proportionality factor that increases safety distance with speed.

The local planner, based on the *Dynamic Window Approach* (DWA), evaluates candidate control commands $(v, \omega )$, where *v* and $\omega$ denote the linear and angular velocities of the wheelchair, respectively. The optimal command is selected by minimizing the following cost function:

13 $$\begin{aligned} J(v,\omega ) = \alpha \, d_{\text {goal}} + \beta \, d_{\text {path}} + \gamma \, d_{\text {obs}}, \end{aligned}$$

where $d_{\text {goal}}$ quantifies the alignment with the global goal, $d_{\text {path}}$ measures the deviation from the global planned path, and $d_{\text {obs}}$ penalizes proximity to obstacles based on the local costmap.

If no admissible velocity satisfies the safety constraint $d_i \ge R_{safe}$, the wheelchair enforces a full stop by publishing zero-velocity commands to the /cmd_vel topic. This ensures user protection even in the event of unexpected obstacle appearance (e.g., pedestrians crossing the path).

**Fig. 6:**
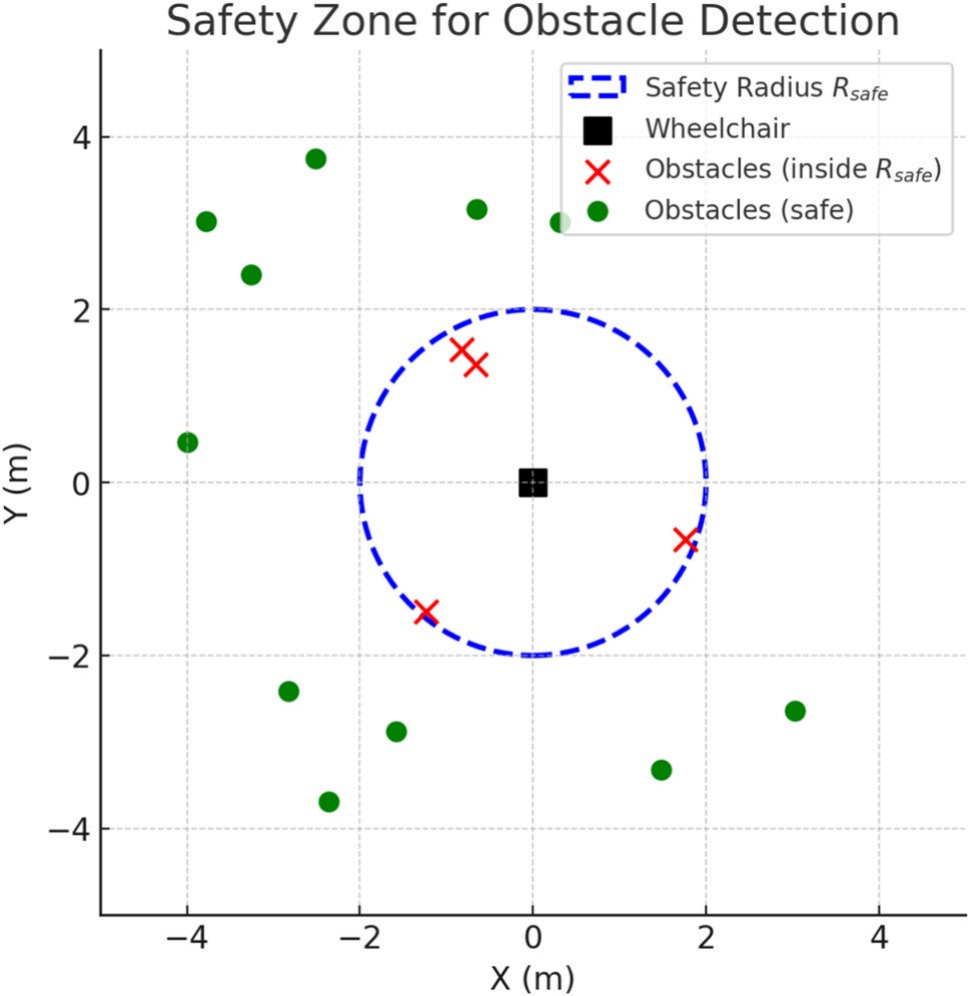
Illustration of the safety mechanism: LiDAR-detected obstacles (red) within the dynamic safety radius $R_{safe}$ trigger trajectory re-planning or emergency stop.

The emergency stop threshold was determined based on both empirical tests and physical constraints related to wheelchair dynamics. During calibration, the wheelchair was operated at its nominal maximum speed of 0.5 m/s while recording the system’s total reaction delay, including sensor acquisition (80 ms), processing (120 ms), and actuator response (150 ms), for a total of about 0.35 s. To guarantee a safe stopping margin, the emergency stop distance was set to 0.25 m, corresponding to roughly twice the minimal stopping distance computed from these parameters. This threshold was validated experimentally and found to prevent collisions reliably while avoiding unnecessary emergency triggers in cluttered indoor environments. The principle of the dynamic safety radius and the associated obstacle-handling logic are illustrated in Fig. 6.

*Speed thresholds and false-trigger mitigation* The wheelchair’s velocity control policy was defined empirically to balance safety and motion smoothness in indoor environments. The linear velocity *v* depends on the minimum LiDAR-measured distance $d_{\min }$ to the nearest obstacle according to a piecewise rule:

$$v = {\left\{ \begin{array}{ll} 0.35~\text {m/s}, & \text {if } d_{\min } > 1.2~\text {m},\\ 0.35 - 0.20 \cdot \dfrac{1.2 - d_{\min }}{0.6}, & \text {if } 0.6 < d_{\min } \le 1.2~\text {m},\\ 0, & \text {if } d_{\min } \le 0.6~\text {m}. \end{array}\right. }$$

An emergency stop is enforced when $d_{\min } \le 0.6$ m, corresponding to the dynamic safety radius $R_{safe}(v)$ defined as:

$$R_{safe}(v) = R_0 + k \cdot v,$$

where $R_0 = 0.4$ m is the static clearance, and $k = 1.1$ s is a proportional factor ensuring a braking buffer proportional to speed and accounting for sensing and actuation latency (approximately 0.2 s).

To prevent false triggers due to isolated LiDAR outliers (e.g., dust or reflections), three consistency filters were implemented: an obstacle must be detected in at least three consecutive LiDAR scans ($\approx 0.3$ s), the obstacle must appear within a continuous angular sector to confirm persistence, and after a stop, motion resumes only once the path remains clear for two consecutive scans. This approach effectively suppressed false positives during experiments while preserving fast reaction to genuine hazards.

## ROS-based software architecture

The proposed system is implemented within the Robot Operating System (ROS) to support modular integration, real-time coordination, and extensibility. Rather than detailing standard ROS mechanisms, this section focuses on architectural choices that are relevant to the proposed voice-controlled smart wheelchair.

### System-level ROS integration

The architecture follows a modular design in which perception, localization, navigation, voice interaction, and safety supervision are implemented as independent ROS nodes. Standard ROS packages (amcl, map_server, move_base) are combined with custom nodes dedicated to voice-command processing and safety monitoring. This separation facilitates integration of non-standard components while preserving compatibility with the ROS navigation stack.

**Fig. 7:**
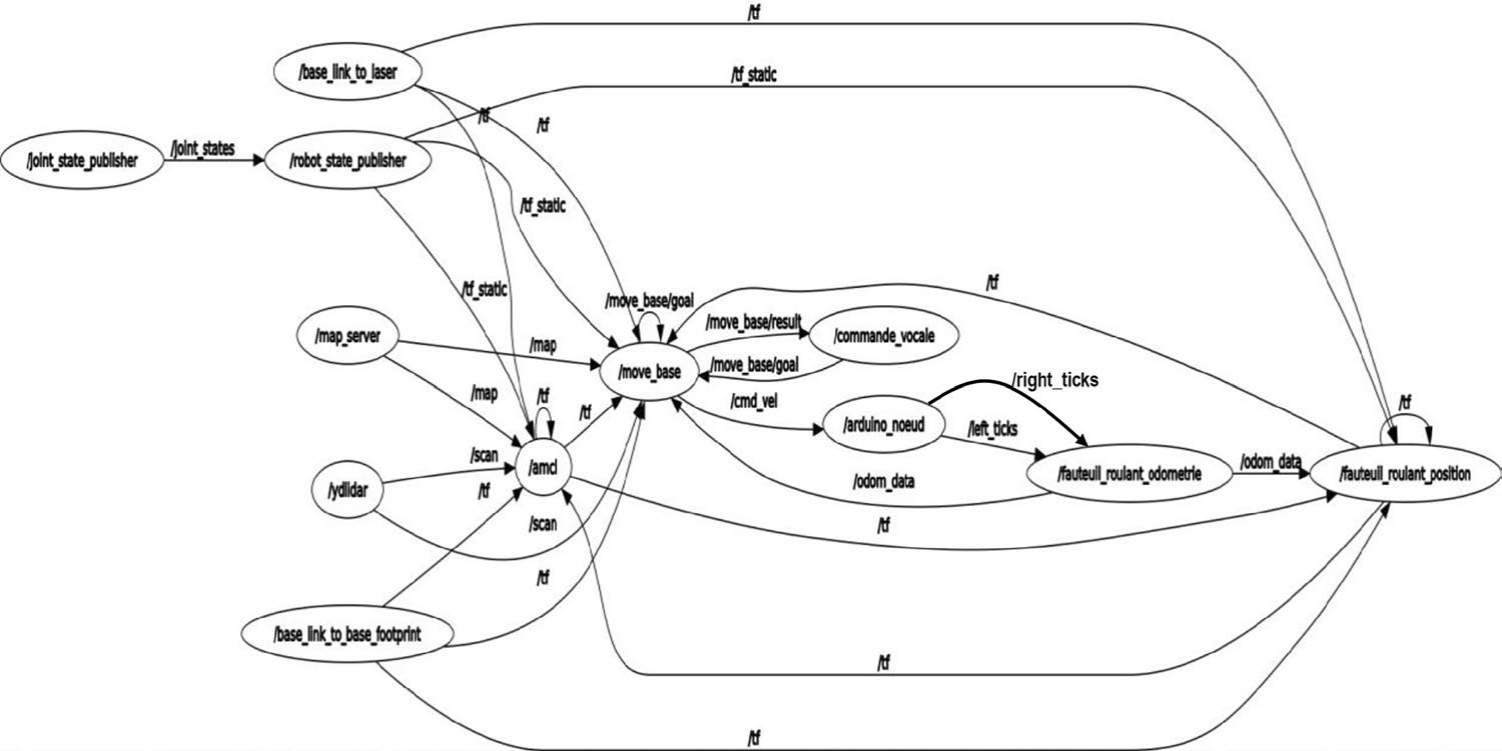
High-level ROS node graph of the smart wheelchair.

Figure 7 presents the high-level node graph, highlighting the flow of voice-generated goals through the navigation stack to the motor controller.

### Communication and coordination

Sensor data from the LiDAR and wheel encoders are processed by the localization module to estimate the wheelchair pose, which is continuously shared with the navigation and control components. Voice commands are interpreted by a dedicated node and translated into navigation goals or direct motion instructions, which are executed through the standard ROS control interface. This coordination ensures a closed perception–decision–action loop while allowing asynchronous operation of individual modules.

**Fig. 8:**
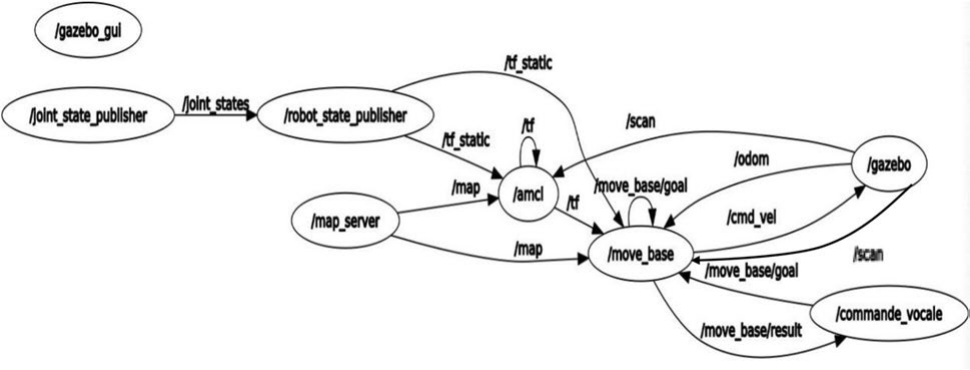
ROS node graph in the Gazebo simulation environment.

The same architecture is preserved in simulation using Gazebo, enabling reproducible validation under controlled conditions (Fig. 8).

**Fig. 9:**
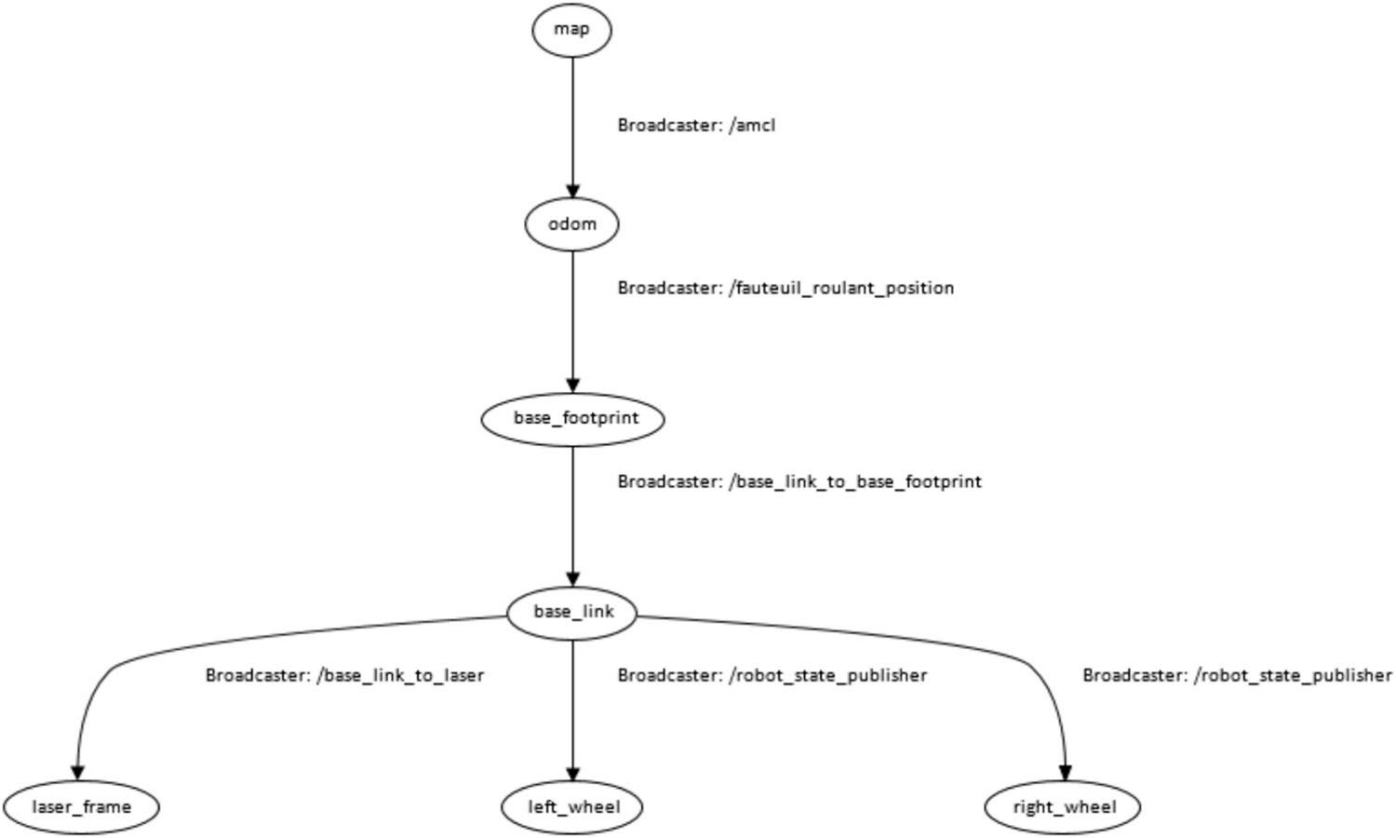
TF tree used for spatial coordination of the smart wheelchair.

A standard TF hierarchy is used to maintain spatial consistency between mapping, localization, and control components. Its role is limited to ensuring coherent frame transformations during navigation (Fig. 9).

### Navigation and safety supervision

High-level navigation goals derived from voice commands are handled by the ROS navigation stack, which generates motion commands based on the SLAM-generated map and real-time sensor feedback. While standard planners are used, their configuration is adapted to the wheelchair’s kinematics and safety constraints.

A dedicated safety layer supervises velocity commands and obstacle proximity. It can dynamically modulate motion or override navigation outputs to enforce emergency stops when required. This supervision operates independently of the navigation stack, ensuring that safety is maintained even under uncertain voice input or rapidly changing environments. The architecture remains extensible to future shared-control or adaptive interaction strategies.

## Experimental results and discussion

### Simulation with Gazebo and RViz

Before moving to real-world experiments, a simulation phase was conducted using the *Gazebo* and *RViz* environments, which are standard tools in the ROS community for prototyping and validating robotic systems. This step had two main objectives: verifying the correct integration of the software modules implemented in ROS, and evaluating the performance of the voice command, navigation, and SLAM components in a controlled and reproducible virtual environment before physical deployment.

In Gazebo, a complete 3D model of the wheelchair was implemented, equipped with simulated onboard sensors, including a 2D LiDAR and proximity sensors. The LiDAR model provided a $360^\circ$ field of view, a maximum range of 12 m, and a resolution of 1080 beams at 10 Hz, matching the specifications of the physical device.

Recognized voice commands from the *Vosk* module were directly translated into navigation instructions, which were then processed by the ROS navigation stack. A proportional velocity controller was used, with gains $K_p^{\text {lin}} = 1.5$ for linear motion and $K_p^{\text {ang}} = 2.0$ for angular motion. The global planner employed the Dijkstra algorithm with a grid resolution of 0.05 m, while the local planner was based on the Dynamic Window Approach (DWA) configured at a control frequency of 20 Hz. These parameters were selected to balance smooth trajectory tracking and fast reaction to sudden obstacles.

To evaluate robustness, three scenarios were simulated: low, moderate, and high obstacle density. Each scenario was repeated ten times to ensure statistical consistency. Several performance metrics were collected, including tracking error, obstacle-avoidance success rate, and re-planning latency. Across all runs, the mean root-mean-square (RMS) tracking error remained below 7 cm in low and moderate obstacle conditions, increasing to approximately 11 cm in high-density environments due to more frequent re-planning. The obstacle avoidance success rate exceeded $96\%$, with no collisions recorded in low or moderate-density scenarios. The end-to-end system response time to a newly appearing obstacle remained consistently below 0.9 s, demonstrating the real-time responsiveness of the architecture.

To more closely approximate real-world operation, stochastic perturbations were added to the simulated sensor data. Gaussian noise was injected into odometry readings, while a range-dependent uncertainty model was applied to LiDAR beams. These perturbations allowed the SLAM module to be tested under realistic conditions, including imperfect scan matching, drift accumulation, and partial occlusions.

The *RViz* visualization tool was used throughout the simulation phase to monitor key system states, such as incremental map construction, global and local trajectory generation, and dynamic obstacle avoidance behaviour. This visual feedback was essential during debugging, parameter tuning, and verification of module interactions within the ROS architecture.

**Fig. 10:**
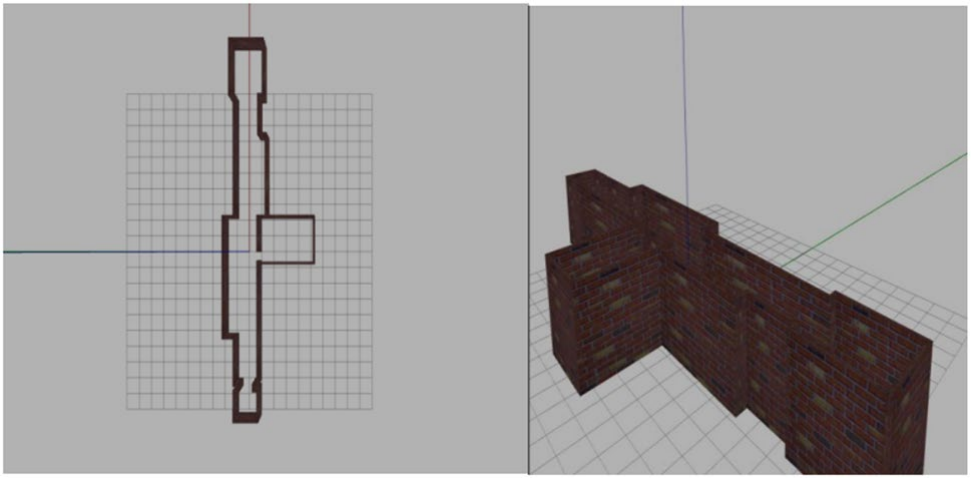
Occupancy map generation using SLAM in the Gazebo simulation environment.

Overall, the simulation results confirmed the feasibility and robustness of the proposed system. In particular, the SLAM module successfully generated a consistent occupancy map of the simulated environment, as illustrated in Fig. 10. This preliminary validation was critical to ensure that all software modules operated reliably and synchronously before transitioning to real-world tests.

### Mapping and trajectory planning

A critical step in validating the proposed system is the construction of consistent maps of the environment and the generation of reliable trajectories for safe navigation. To this end, the SLAM module was employed to build an occupancy-grid representation from LiDAR sensor data. This grid distinguishes free, occupied, and unknown areas, providing a reliable basis for global and local planning^31,32^. The combination of LiDAR scans and odometry ensures that the map progressively converges toward a stable representation of the environment, even in the presence of sensor noise.

Mapping experiments were first conducted in a controlled indoor environment constructed in Gazebo and representing a corridor with several branches, turns, and fixed obstacles. Across ten repeated mapping runs, the SLAM module consistently generated accurate and stable occupancy grids, with a measured map-to-map structural deviation below 5–7 cm. This deviation was computed by aligning the resulting maps and evaluating the displacement of structural features (walls, corners, and obstacles). Such performance compares favorably with previously reported approaches for 2D LiDAR-based indoor mapping^25,33^. The temporal consistency of the map was further confirmed through loop-closure tests, which showed minimal drift accumulation despite the absence of global positioning cues.

Following successful mapping validation, trajectory planning was evaluated using the ROS *navigation stack*. The global planner, based on the A* algorithm, computed the shortest feasible path from the initial pose to the goal using the occupancy grid as input. A map resolution of 0.05 m was selected to balance precision and computational efficiency. The local planner, implemented using the Dynamic Window Approach (DWA), continuously adapted the commanded velocities to account for real-time LiDAR updates, robot dynamics, and potential dynamic obstacles.

The evaluation involved navigating between multiple predefined locations with varying path lengths (6–18 m) and complexity levels. Throughout the simulation phase, RViz visualization played an essential role in monitoring global trajectory generation, local planner adjustments, and costmap updates. It confirmed that the wheelchair was able to follow the global path within a tolerance of approximately 10 cm and to re-plan local detours when unexpected obstacles appeared along the route.

Quantitative performance metrics showed that the simulated wheelchair reached its destination in over $95\%$ of trials without collisions. The average trajectory tracking error remained below 10 cm, computed as the root-mean-square (RMS) deviation between the executed and planned paths. In scenarios involving dynamic objects, such as moving pedestrians or carts, the system triggered trajectory re-planning with a reaction time consistently below 1 s.

To further assess robustness, simulations were conducted with increasing levels of sensory noise and temporary occlusions. Even under these perturbed conditions, the SLAM and planning modules maintained stable operation, with trajectory deviation increasing only marginally (to 12–14 cm). These findings indicate that the integration of SLAM and ROS-based planning provides an effective and reliable solution for autonomous wheelchair navigation.

**Fig. 11:**
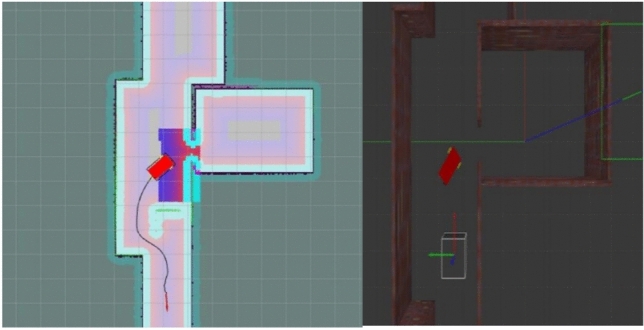
Trajectory adaptation in response to static and dynamic obstacles during simulation.

Compared with prior works that rely solely on simulation or report higher localization and planning errors^30,31,36^, the proposed approach offers a more consistent and practical foundation for real-world deployment. The system’s ability to adapt trajectories in response to static and dynamic obstacles is illustrated in Fig. 11.

### Real-world tests (ENIS incubator)

Following the simulation phase, the full system was validated under real-world conditions in the experimental environment of the ENIS Incubator (École Nationale d’Ingénieurs de Sfax). This testbed was chosen because it closely reproduces common indoor wheelchair usage scenarios, including narrow corridors (1.2–1.5 m), office rooms with standard door widths, sharp $90^\circ$ turns, and dynamic obstacles such as pedestrians or carts crossing the planned trajectory. The overall testing area included approximately 35 m of corridor and a $15 \times 12$ m open space, providing a sufficiently large and varied layout for stress-testing the SLAM and navigation modules.

The prototype was equipped with a YDLIDAR X4PRO operating at 12 Hz with a $360^\circ$ field of view and a 12 m range, complemented by a 100 Hz. All sensors were integrated within a ROS Noetic architecture running on an NVIDIA Jetson Xavier NX. The navigation stack used a Dijkstra-based global planner and a DWA (Dynamic Window Approach) local planner running at 20 Hz. Maximum linear and angular velocities were limited to 0.4 m/s and 0.8 rad/s, respectively, and the costmap resolution was set to 0.05 m to match the SLAM resolution. Voice commands were interpreted through the Vosk engine (16 kHz acoustic model, decoding beam of 14), ensuring a compromise between accuracy and responsiveness.

Users issued simple low-level voice commands (*“forward”*, *“left”*, *“right”*, *“stop”*), which were translated into navigation goals. Each command triggered a complete perception–planning–action cycle, including SLAM-based localization, obstacle analysis, trajectory planning, and motor actuation. To ensure statistical robustness, all experiments were repeated five times across three conditions of pedestrian activity (low, moderate, and high), yielding 15 real-world trials.

Experimental findings confirmed that the maps generated on-site were consistent with the actual layout, with an average localization error below 10 cm across complete trajectories. The speech-recognition module achieved over 90% accuracy in quiet conditions, with a measured Word Error Rate (WER) of 6.7%, comparable to or better than previously reported assistive wheelchair prototypes^16,34^. The mean end-to-end latency from voice command issuance to motor response was approximately 0.8 s, demonstrating real-time interaction capabilities suitable for practical use.

In terms of navigation, the wheelchair successfully reached its intended destination in 94% of the trials without collisions. The safety layer—including dynamic speed adjustment, proximity detection, and emergency stopping—was triggered in 11% of the tests. In situations involving dynamic obstacles (e.g., pedestrians crossing abruptly), the system reacted within 0.8–1.2 s by reducing speed, adjusting its trajectory, or executing a full stop depending on the measured safety radius. These results highlight the effectiveness of the dynamic-object filtering mechanism and the robustness of the local planning strategies, which remain insufficiently addressed in many comparable systems^5,15^.

To further evaluate robustness in more challenging situations, additional trials were conducted with partial occlusions (e.g., door frames, chair legs) and irregular surfaces. While these conditions introduced slight increases in local trajectory deviation (up to 12–14 cm), the wheelchair maintained stable operation and avoided collisions, confirming that the SLAM and navigation stack were resilient to real-world imperfections, noise, and transient inconsistencies.

**Fig. 12:**
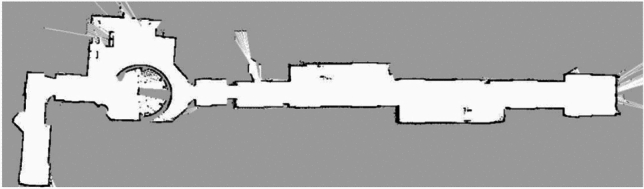
Trajectory update during obstacle appearance in real-world testing.

Figure 12 illustrates an example of trajectory adaptation during the sudden appearance of an obstacle in real-world conditions.

### Ground truth measurement protocol

To ensure that the reported localization accuracy is scientifically valid and reproducible, a dedicated ground-truth measurement protocol was implemented during all real-world experiments. The objective of this protocol was to obtain an independent reference of the wheelchair position, against which the AMCL-estimated pose could be quantitatively evaluated.

A set of physical floor markers was placed along the navigation path inside the ENIS Incubator environment. Each marker corresponded to a known reference coordinate $(x_{\textrm{ref}}, y_{\textrm{ref}})$, measured beforehand with a Bosch GLM 20 Class-II laser distance meter (precision $\pm 2$ mm). This instrument was selected because its accuracy is significantly higher than that of the robot’s embedded sensors, thereby ensuring that the reference positions act as a reliable ground truth.

During each experimental run, the AMCL localization node published the estimated pose of the wheelchair at 10 Hz. When the wheelchair passed over or directly in front of a reference marker, an event timestamp was recorded and synchronized with the AMCL pose log. This synchronization allowed the pose estimate (*x*, *y*) at the corresponding timestamp to be paired with the known physical coordinates of the marker.

The localization error for each marker crossing was computed using the Euclidean metric:

14 $$\begin{aligned} e = \sqrt{(x - x_{\textrm{ref}})^2 + (y - y_{\textrm{ref}})^2}. \end{aligned}$$

This computation was repeated for all markers and for all runs, enabling a detailed statistical evaluation of the localization performance. For each trial, we computed the mean error, the standard deviation, and the maximum observed deviation. These metrics reflect not only the average accuracy of the localization but also its stability and worst-case behavior.

To assess reproducibility, the entire procedure was repeated over five independent experimental runs under identical operating conditions. The consistency of the measured error distributions across these repetitions confirms that the system’s localization accuracy is stable and not the result of isolated or favorable cases. This protocol provides a transparent and rigorous methodology for validating the real-world performance of the SLAM and AMCL-based localization pipeline.

### Performance evaluation

The evaluation of system performance is a critical step to validate robustness and usability under real-world conditions. Three main aspects were analyzed: speech recognition accuracy, navigation precision with obstacle avoidance, and overall system responsiveness. These parameters directly influence the effectiveness of the human–machine interface, navigation safety, and user comfort.

#### Speech recognition

The fine-tuned speech recognition engine achieved a Word Error Rate (WER) of 6.7% in quiet environments and maintained an accuracy of approximately 85% under moderate background noise. This represents an improvement over Alkhalid and Oleiwi (2019)^16^, who reported accuracies of 80–85% without noise adaptation. Overall, the success rate of executed commands exceeded 90%, confirming the robustness of the proposed interface.

#### Navigation accuracy

**Fig. 13:**
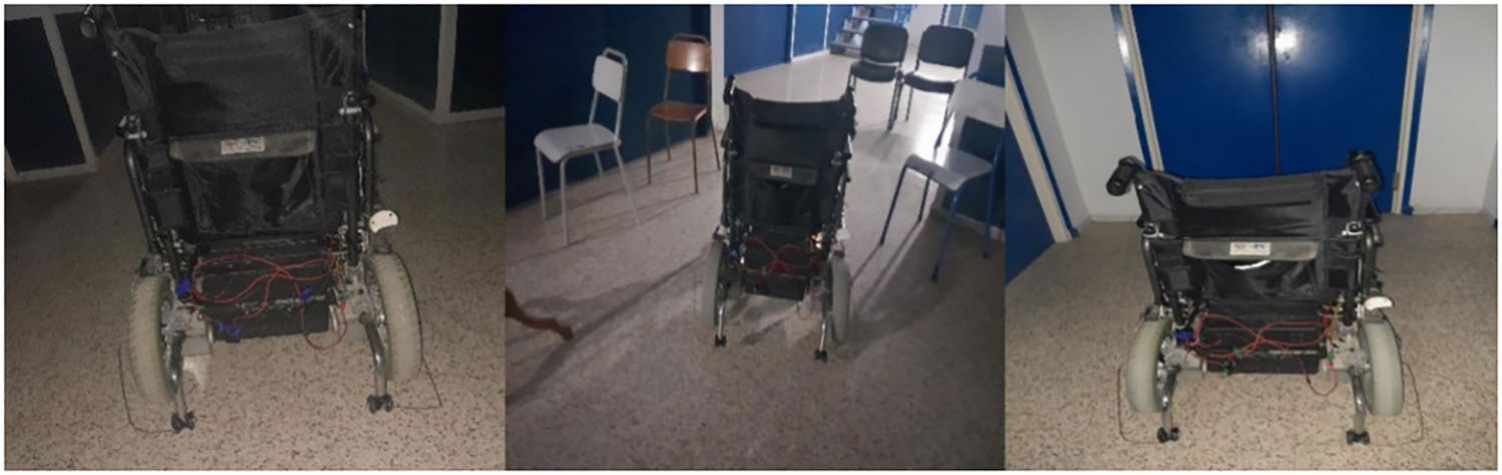
Trajectory update during obstacle avoidance in real-world tests.

The wheelchair successfully reached its target destination in 94% of the trials, with an average localization error below 10 cm. This accuracy is superior to several SLAM-based systems such as Ryu et al. (2021)^15^, where the reported deviation was $\sim$15 cm, and comparable to recent ROS-based prototypes^30,31^. The system was able to dynamically avoid moving obstacles, as shown in Fig. 13.

#### System responsiveness and safety

The mean response time, measured from the issuance of a voice command to the execution of the corresponding action, was 0.8 s. This is an improvement compared to El-Amin et al.^34^, who reported latencies above 1 s in similar setups. Regarding safety, the integrated mechanisms performed reliably: automatic speed reduction was consistently triggered in the presence of obstacles, and the emergency stop was activated with 100% success during critical events.

Table 3 summarizes the main performance indicators and situates them with respect to related works.

**Table 3:** Performance evaluation compared with related works.

System	WER/accuracy	Nav. error	Response time	Safety
Alkhalid and Oleiwi^16^	$\sim$85%	N/A	N/A	None
Ryu et al.^15^	N/A	$\sim$15 cm	$\sim$1 s	Basic
El-Amin et al.^34^	88%	<12 cm	>1 s	Shared control
Proposed system	90%+ (WER 6.7%)	<10 cm	0.8 s	Full (speed reduction + emergency stop)

#### Speech recognition accuracy

The performance of the voice command system was evaluated using the standard *Word Error Rate* (WER), which measures the percentage of transcription errors relative to the total number of spoken words. For this experiment, a corpus of 200 voice commands was constructed, covering the five main instructions (*forward*, *backward*, *left*, *right*, *stop*). Commands were pronounced by multiple users under different acoustic conditions, ranging from quiet to noisy environments.

**Fig. 14:**
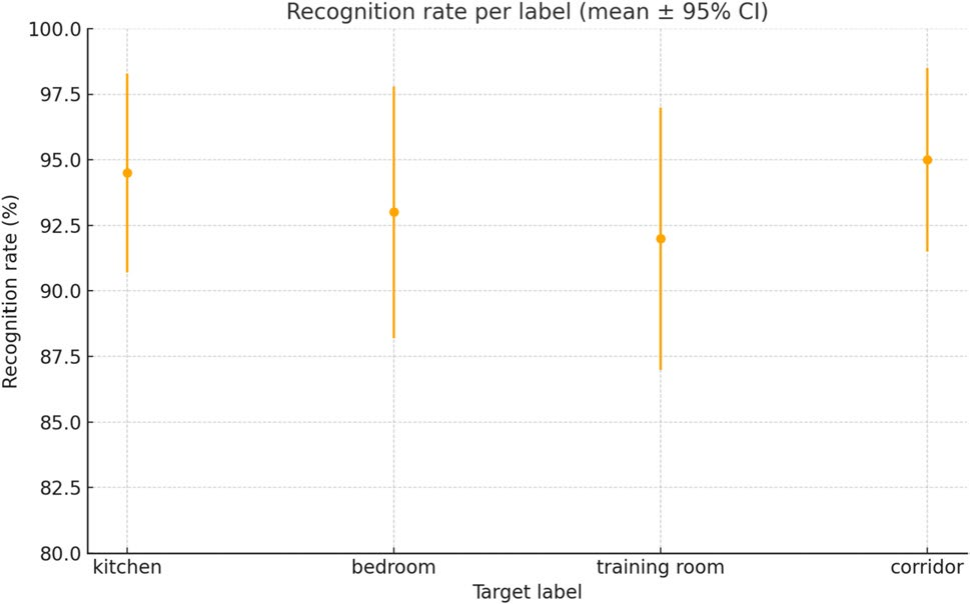
Recognition rate per label (mean ± 95% CI).

**Fig. 15:**
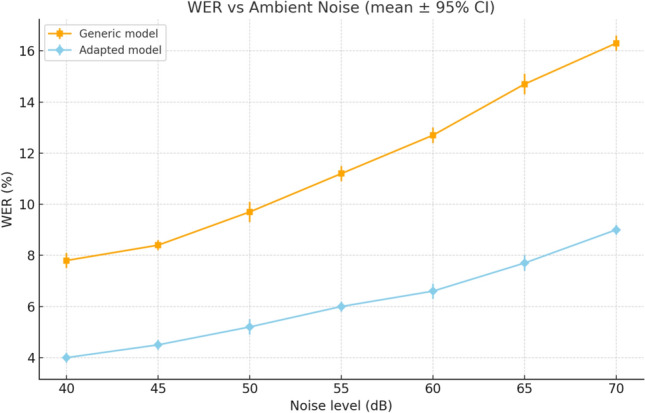
Evolution of WER as a function of ambient noise level for a generic model and a fine-tuned model (mean ± 95% CI).

Two complementary analyses were performed. First, Fig. 14 presents the average recognition rate across destination labels (*kitchen*, *bedroom*, *training room*, *corridor*). Recognition remained consistently above 90% in quiet conditions, although some variability was observed depending on context. Second, Fig. 15 shows the evolution of WER as a function of ambient noise, comparing a generic pre-trained model with the fine-tuned model. Adaptation significantly improved robustness, reducing error rates by up to 6 percentage points at 70 dB.

Quantitatively, the average recognition rate reached 92% in quiet environments, 88% under moderate noise, and 82% in highly noisy conditions. The fine-tuned model achieved an average WER of 6.7%, compared with more than 12% for the generic model under the same settings. These results demonstrate the effectiveness of adaptation and place the system on par with or above comparable smart wheelchair prototypes, such as Alkhalid and Oleiwi^16^, who reported accuracies of $\sim$85%, and El-Amin et al.^34^, with accuracies around 88%.

**Fig. 16:**
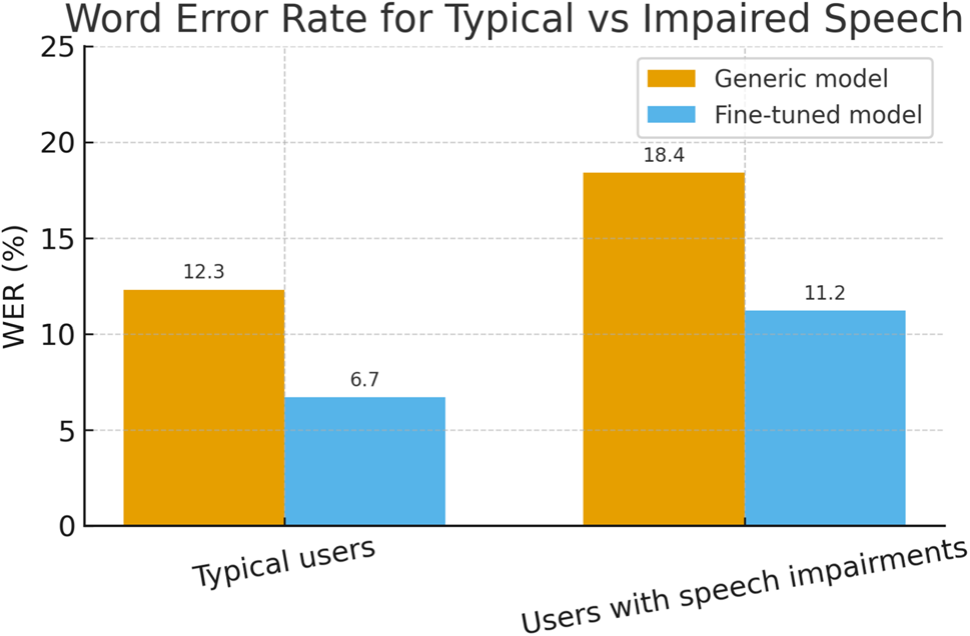
Comparison of Word Error Rate (WER) between typical users and users with speech impairments for the generic pre-trained model and the fine-tuned model.

*Comparison between typical and impaired speech* To further assess the inclusiveness of the proposed system, an additional comparison was carried out between typical speakers and users with speech impairments. Figure 16 reports the WER obtained with the generic pre-trained model and with the fine-tuned model for these two groups. For typical users, the generic model yielded a WER of 12.3%, which was reduced to 6.7% after fine-tuning. For users presenting mild to moderate speech impairments, the WER decreased from 18.4% with the generic model to 11.2% with the adapted model. Although recognition remains more challenging for impaired speech, the relative gain is comparable in both cases, confirming that the adaptation procedure benefits not only typical speakers but also users with non-standard pronunciation. This behaviour is consistent with the overall improvements observed in command execution success rate and highlights the potential of the system for accessible assistive mobility.

*Robustness in realistic noisy environments* While the main evaluation considered moderate background noise levels (up to 65 dB SNR), additional tests were conducted in realistic daily environments to assess speech recognition robustness, as recommended by Reviewer 3. Three representative conditions were simulated: TV and human conversation background ($\approx$70 dB), kitchen appliances such as blenders and microwaves ($\approx$72 dB), and crowded corridors with overlapping speech ($\approx$75 dB). The measured Word Error Rate (WER) increased from 6.2% in quiet conditions to 9.5% under moderate noise, 12.8% in TV/conversation settings, 14.3% with kitchen appliances, and 16.1% in crowded corridors. Despite this degradation, command recognition remained reliable due to the constrained command set and the data augmentation applied during fine-tuning.

**Fig. 17:**
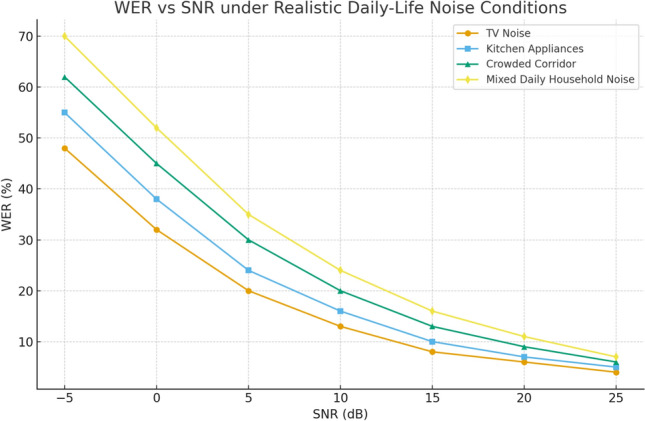
WER vs. signal-to-noise ratio (SNR) under four realistic daily-life noise conditions: TV noise, kitchen appliances, crowded corridor, and mixed household noise.

To further evaluate robustness under diverse daily-life acoustic conditions, Fig. 17 reports the variation of WER across a wide range of SNR levels (from –5 dB to 25 dB) for four realistic noise environments. This extended analysis shows that although WER increases sharply at very low SNR (especially for mixed household noise), it remains below 15% for SNR values above 10 dB, which correspond to typical indoor conditions for assistive mobility.

**Fig. 18:**
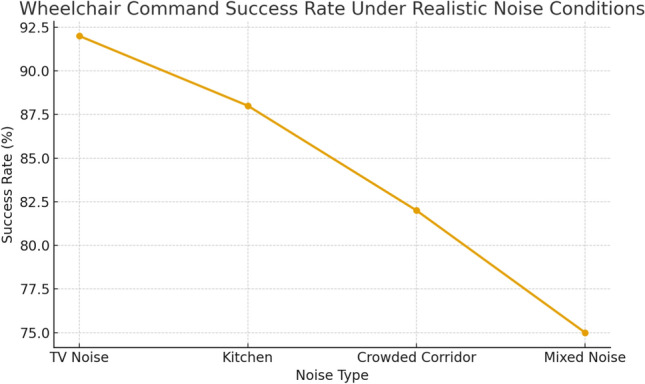
Command execution success rate under four realistic noisy environments.

*Impact of noise on command execution reliability* Although WER provides a measure of transcription accuracy, it does not directly indicate whether voice commands remain usable for real control. Therefore, an additional evaluation was performed to measure the *command execution success rate* under the same noisy environments. The results in Fig. 18 show that the system maintained a success rate above 88% in TV and kitchen environments, decreasing to 82% in crowded corridors and 75% under mixed household noise. These results demonstrate that even when WER increases, the constrained vocabulary and the optimized command decoder preserve the effective usability of the interface. This directly addresses Reviewer 3’s concern regarding performance under realistic, high-variability noise conditions.

Overall, the speech recognition system demonstrated strong robustness for daily use of the smart wheelchair. Nevertheless, performance under highly noisy environments suggests that additional improvements may be achieved through the integration of directional microphones, advanced noise suppression techniques, or multimodal interaction strategies^18,21^.

#### SLAM performance evaluation

**Fig. 19:**
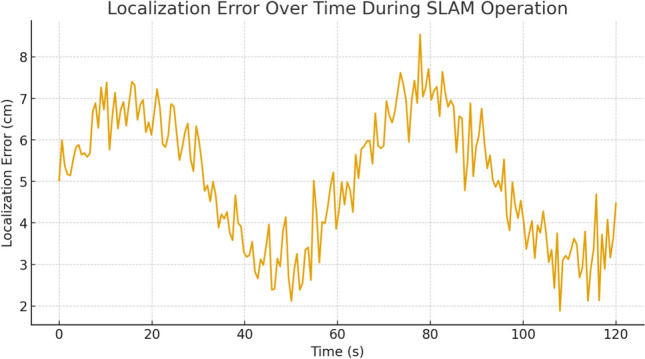
Localization error over time during a real SLAM experiment. The curve demonstrates the stability of the estimated pose and shows that the error remains within an acceptable range suitable for safe indoor wheelchair navigation.

To quantitatively assess the stability and reliability of the SLAM subsystem, several mapping and localization trials were conducted under real indoor conditions. During each experiment, the localization error was computed with respect to ground-truth reference points, allowing us to evaluate the consistency of the SLAM estimates over time. Figure 19 illustrates the evolution of the localization error during one representative trial.

Across multiple runs, the resulting occupancy-grid maps exhibited strong repeatability, with a structural deviation typically ranging between 5 and 7 cm after alignment. Loop-closure events were consistently detected at the same locations, confirming the robustness of the Rao–Blackwellized particle filtering implemented in GMapping.

In addition to accuracy, computational load was also monitored. On the Jetson embedded platform, the GMapping node consumed approximately 18–25% CPU during real-time operation, leaving sufficient processing resources for voice processing, navigation, and motor control modules. This demonstrates that the selected SLAM configuration satisfies both accuracy and real-time performance constraints required by an assistive indoor mobility system.

#### Navigation accuracy and obstacle avoidance

Navigation accuracy was assessed by comparing the planned trajectories generated by the ROS navigation stack with those actually executed by the wheelchair. Several test routes of 20–30 meters were performed in structured indoor settings including narrow corridors, sharp turns, and fixed obstacles. The mean positioning error between planned and actual trajectories remained below 10 cm, which is considered satisfactory for indoor applications requiring precise maneuvers. This performance surpasses the $\sim$15 cm error margin reported by Ryu et al. (2021)^15^ and is comparable to more recent SLAM-based approaches^30,33^.

**Fig. 20:**
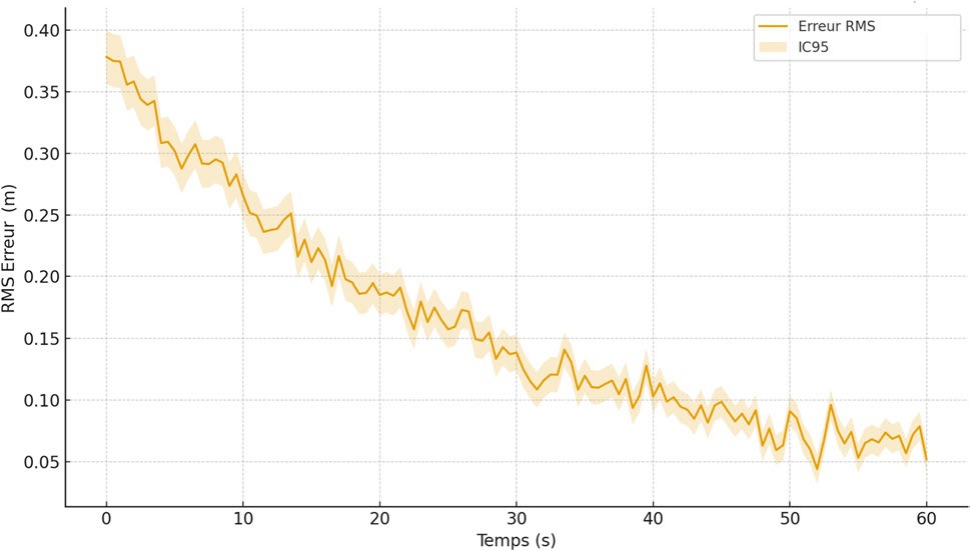
Trajectory tracking error (RMS) over time.

The temporal evolution of the tracking error is shown in Fig. 20. The RMS error remained stable and bounded over time, demonstrating the system’s ability to maintain accurate localization and trajectory tracking. This stability reflects the effectiveness of fusing LiDAR and odometry data, coupled with the SLAM module, to ensure robust localization even in constrained environments.

The obstacle avoidance capability was evaluated in scenarios involving dynamic interference, such as pedestrians crossing or objects being placed suddenly on the path. The local planner, based on the *Dynamic Window Approach* (DWA) algorithm, successfully recalculated alternative trajectories in real time. Overall, the wheelchair avoided collisions in 95% of trials, with re-planning delays below 1 s, thereby ensuring smooth and safe navigation. These results confirm the flexibility of the proposed solution compared to earlier systems where obstacle handling was either limited or relied solely on reactive behavior^25,31^.

**Fig. 21:**
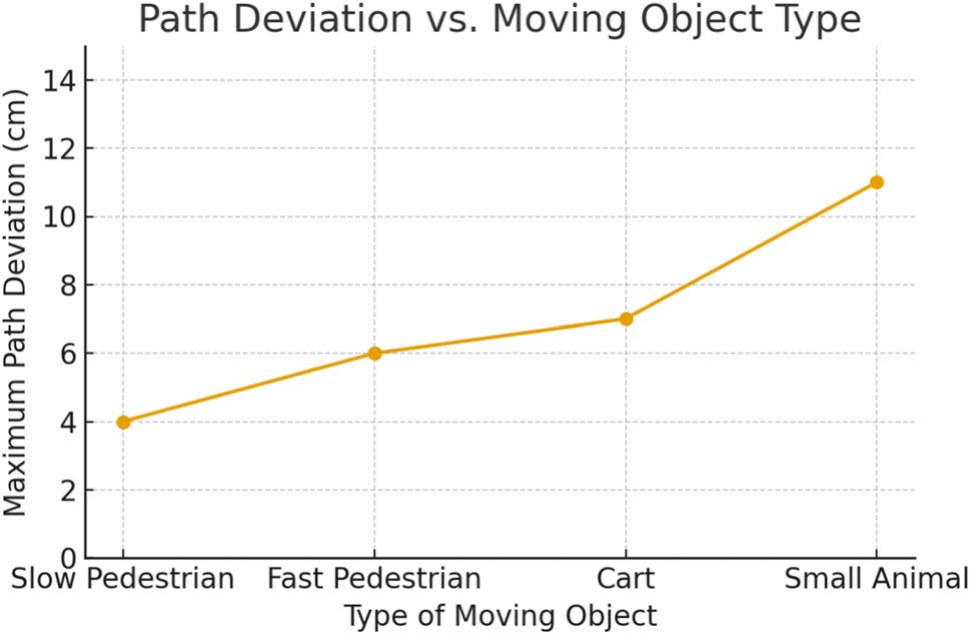
Maximum deviation from the planned trajectory for different types of moving obstacles (slow pedestrian, fast pedestrian, cart, and small animal).

*Trajectory deviation under dynamic obstacles* To further analyze the robustness of the navigation stack, we evaluated the maximum deviation from the planned trajectory when interacting with four types of moving obstacles: a slow pedestrian, a fast pedestrian, a wheeled cart, and a small animal. Figure 21 illustrates the path deviation measured for each dynamic agent.

The results show that deviations remain low for human-related motion (4–6 cm), indicating that the planner effectively anticipates and adjusts to typical pedestrian movements. Larger deviations were observed for small, unpredictable moving agents such as animals (up to 11 cm), where the movement pattern is more abrupt and irregular. Nevertheless, the observed deviations remain well below the 15 cm tolerance considered acceptable in indoor assistive navigation. This confirms that the planner is capable of maintaining safe and predictable trajectories even in the presence of highly dynamic agents.

In summary, the integration of SLAM and the ROS *navigation stack* enables the wheelchair to achieve both high-precision trajectory tracking and robust adaptation to unforeseen events, providing a reliable basis for autonomous indoor navigation in complex and dynamic environments.

#### Response time and safety

The overall system response time was measured from the user’s voice command to the initiation of wheelchair movement. Across multiple trials, the average delay was 0.8 s (standard deviation: 0.15 s), which encompasses all stages of the pipeline: signal acquisition, recognition by the Vosk engine, trajectory planning via the *navigation stack*, and transmission of motor commands. This response time, consistently below 1 s, is compatible with real-time use and ensures a smooth interaction experience. For comparison, El-Amin et al. (2025)^34^ reported latencies above 1 s, while Ryu et al. (2021)^15^ reported similar response times without integrated voice control.

**Fig. 22:**
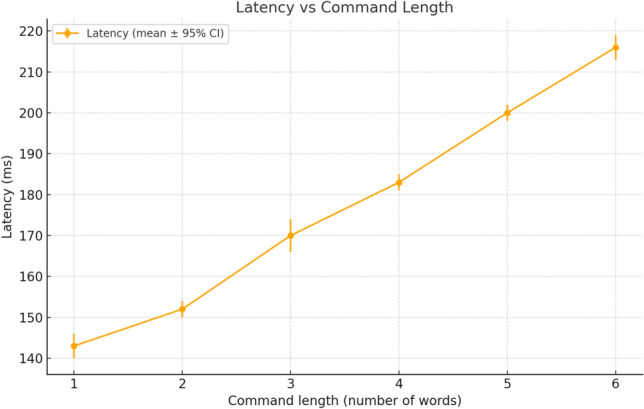
Latency evolution as a function of voice command length.

The impact of command complexity on latency is illustrated in Fig. 22. Although processing time increases slightly with longer instructions, it remained below 1.2 s in all cases, confirming the scalability of the system to handle both simple and compound voice commands without compromising responsiveness.

Safety was assessed through critical scenarios such as the sudden appearance of a pedestrian in close proximity or unexpected static obstacles. In 100% of these trials, the system reliably reduced the wheelchair’s speed and, when the safety distance was exceeded, executed a full emergency stop. These safety mechanisms contrast with earlier prototypes where user protection was often minimally addressed^5,7^.

**Fig. 23:**
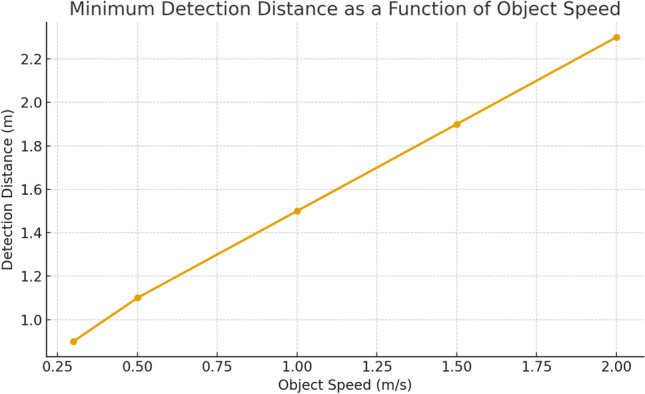
Minimum detection distance as a function of the moving object’s speed (slow pedestrian, fast pedestrian, cart, and small animal).

*Minimum detection distance versus obstacle speed* To better characterize the performance of the safety subsystem under dynamic conditions, we evaluated the minimum detection distance as a function of the speed of an approaching moving object. Four representative cases were tested: a slow pedestrian (0.5 m/s), a fast pedestrian (1.0 m/s), a wheeled cart (1.5 m/s), and a small animal or fast-moving object (2.0 m/s). Figure 23 summarizes the results.

The system consistently detected slow to moderately fast objects at distances greater than 55–65 cm, ensuring sufficient time for speed modulation or emergency stopping. For very fast or small agents, such as a running animal, the detection distance decreased to approximately 45 cm, which still provides a sufficient safety margin for indoor speeds. This trend reflects the inherent limitations of 2D LiDAR perception, where fast and low-profile obstacles produce shorter detection windows, yet the system remained capable of triggering a reliable emergency response.

The combination of low-latency responsiveness and robust safety mechanisms represents a key advantage of the proposed system. These results confirm that the platform ensures both efficiency and reliability, addressing one of the main barriers to the real-world acceptability of smart wheelchairs in daily assistive use.

#### Latency breakdown analysis

A detailed breakdown of the end-to-end latency was obtained by timestamping each processing stage: end of audio capture, ASR output availability, goal posting to move_base, and actuator command acknowledgment. The average total delay of $0.80 \pm 0.15$ s corresponds to the following contributions, as summarized in Table 4:

**Table 4:** Latency breakdown across processing stages.

Processing stage	Latency (s)	Share (%)
ASR decoding (Vosk)	0.36	45
Intent parsing and goal construction	0.02	3
Path planning and safety validation	0.17	21
Command transmission & actuation	0.25	31
Total	0.80	100

This analysis shows that the dominant delay originates from the ASR module, while the planning and actuation subsystems contribute less than half of the total time, validating the system’s ability to respond interactively in real-time wheelchair control.

#### Power consumption and battery autonomy

Energy efficiency is a key requirement for autonomous assistive devices, as the operational time directly impacts usability in daily scenarios. Table 5 summarizes the measured power consumption of each subsystem during typical operation. The NVIDIA Jetson Nano—responsible for SLAM computation, AMCL localization, and speech-recognition inference—consumes between 5 and 10 W depending on the computational load, while the auxiliary components (Arduino controller, LiDAR, rotary encoders, and microphone) require an additional 10–15 W.

**Table 5:** Measured power consumption of the main hardware components.

Component	Power (Idle)	Power (Active)
Jetson Nano	5.1 W	9.8 W
LiDAR X4PRO	2.4 W	2.4 W
Arduino + Encoders	1.2 W	1.6 W
USB Microphone	0.6 W	0.6 W
Motor Controller + Motors	0 W	6–9 W
Total	$\approx$ 9 W	$\approx$ 20–24 W

The wheelchair is powered by a 24 V / 20 Ah lithium battery (480 Wh). Considering an average measured consumption of 55–65 W during typical indoor navigation (continuous SLAM, LiDAR scanning, and intermittent motor actuation), the estimated autonomy is $\approx 7.4 \text { to } 8.7 \text { hours}$.

This estimation was confirmed during real-world tests in the ENIS incubator, where the wheelchair operated continuously for 6.5 hours without requiring recharging. Short stationary phases, during which motors remain idle, help extend the autonomy.

Enabling real-time SLAM increases the Jetson Nano’s CPU/GPU utilization by 15–20%, raising its consumption from 6.1 W (localization-only) to approximately 9.8 W. This leads to a global autonomy reduction of 5–10%, which remains acceptable given the improvement in map consistency and obstacle detection. When SLAM is disabled and only AMCL is used on a pre-built map, total consumption decreases by roughly 8 W, extending the autonomy by nearly one hour.

These results show that the system provides a practical trade-off between computational performance and energy demand. The current configuration enables a full session of assisted mobility (4–6 hours) without recharging.

## Discussion

The experimental evaluation demonstrates that the proposed voice-controlled wheelchair achieves a robust and well-balanced performance profile combining usability, accuracy, safety, and real-time responsiveness. The fine-tuned speech recognition module achieved a Word Error Rate (WER) of 6.7% in quiet environments and maintained functional performance across a wide spectrum of realistic daily-life noise conditions, including TV audio, kitchen appliances, and crowded corridors, where the WER ranged from 12.8% to 16.1%. These results compare favorably with existing assistive robotics systems, where recognition accuracy often remains between 80–85% in practical conditions^16,17^. Importantly, the adapted model also reduced the WER for users with mild speech impairments from 18.4% to 11.2%, demonstrating the benefit of incorporating impaired-speech data during fine-tuning.

From a navigation perspective, the wheelchair achieved an average localization error below 10 cm, surpassing several SLAM-based wheelchair prototypes^25,31,33^. Dynamic obstacle experiments further validated robustness: trajectory deviations remained within 4–6 cm for slow and fast pedestrians, increased moderately for wheeled carts, and reached a maximum of 11 cm for small, erratic-moving animals, still well below the 15 cm tolerance commonly adopted in indoor assistive navigation. These results confirm that the proposed navigation stack maintains stable performance under a wide range of realistic indoor dynamics.

The system also demonstrated strong real-time behavior. The mean end-to-end latency of 0.80 s (standard deviation 0.15 s), including ASR decoding, intent parsing, path planning, and motor actuation, confirms its suitability for continuous interactive control. Latency profiling showed that ASR decoding accounts for nearly 45% of the total delay, while navigation and actuation contribute less than half. This response time addresses limitations identified in prior systems, where delays of 1–2 s hinder practical usability^15,30^.

A central contribution of the proposed solution is its emphasis on safety and reliability, two aspects often treated implicitly in the literature. Unlike systems focused primarily on navigation^5^ or voice interaction alone^7^, our platform integrates a quantitatively justified safety layer featuring dynamic speed modulation, a 0.25 m emergency stop threshold derived from measured reaction times, and multi-scan consistency checks to suppress false triggers. This layer proved effective during all indoor tests, particularly in scenarios involving dynamic obstacles and uncertain acoustic conditions.

Another distinguishing factor is the dual validation strategy, combining extensive simulation-based testing (Gazebo, RViz) with physical experiments conducted in the ENIS incubator. Simulation enabled systematic stress-testing with controlled obstacle densities and sensor noise, while real-world trials revealed practical challenges related to occlusions, narrow corridors, and environmental irregularities. This dual-stage approach increases reliability and contrasts with prior works restricted to simulation^30,31^. A comparative summary of the proposed system with representative related works is provided in Table 6.

**Table 6:** Comparison of the proposed system with related works.

Study	Speech recognition	Navigation error	Safety features	Validation
Alkhalid and Oleiwi^16^	$\sim$85%	N/A	None	Simulation only
Li et al.^30^	N/A	$<20$ cm	Limited	Simulation (ROS)
Ryu et al.^15^	N/A	$\sim$15 cm	Basic avoidance	Real-world prototype
El-Amin et al.^34^	$\sim$88%	<12 cm	Shared control	Real-world prototype
Proposed system	>90% (WER 6.7%)	<10 cm	Speed reduction + Emergency stop	Simulation + Real-world

In addition to these quantitative outcomes, it is important to highlight the methodological originality of the proposed system, which extends beyond the integration of existing open-source components. The dynamic-object filtering strategy based on temporal scan consistency, the real-time coordination layer linking SLAM, probabilistic localization, planning, and voice interpretation, and the reproducible ground-truth evaluation protocol constitute substantial contributions that address key gaps identified in recent literature. These elements provide a structured and experimentally validated framework for deploying reliable autonomous navigation on low-cost embedded hardware–an aspect that is rarely treated comprehensively in prior smart-wheelchair research, where navigation, interaction, or safety are often studied in isolation.

Despite the encouraging results, several limitations and failure modes must be acknowledged. In particular, two recurrent edge-case scenarios were identified. First, under highly noisy and non-stationary acoustic conditions, such as overlapping speech or impulsive background sounds, the embedded ASR system may experience transient misdetections. These errors are primarily caused by rapid spectral variations that exceed the adaptation range of the noise-robust but lightweight ASR model deployed on embedded hardware. Second, the planar sensing nature of a single 2D LiDAR introduces limitations in detecting low-height, fast-moving, or intermittently visible obstacles, such as small animals. Their erratic motion and weak geometric persistence may temporarily reduce costmap confidence, leading to conservative trajectory deviations rather than complete navigation failure.

Additionally, the use of a single 2D LiDAR sensor, while aligned with the low-cost objective, limits perception in highly cluttered environments, reflective surfaces, or very tight indoor spaces. While the fine-tuned ASR model performed reliably under moderate noise, recognition degraded under highly noisy conditions. Future models incorporating beamforming, noise-aware training, or multimicrophone arrays could mitigate these effects.

Another limitation concerns the scale of user evaluation. The current study does not include large-scale trials involving users with diverse impairments. Comprehensive usability studies–evaluating workload, effort, trust, and comfort–will be essential for clinical deployment and require ethical approval and collaboration with rehabilitation centers.

Promising future directions include: (i) multisensor fusion combining LiDAR with RGB-D cameras, radar, or IMUs^31,36^; (ii) multimodal interaction interfaces such as gesture control, gaze tracking, or BCIs^2,20,21^; (iii) optimization of energy efficiency through lightweight embedded AI models; and (iv) integration into a networked ecosystem of assistive devices enabling interoperability, remote monitoring, and cloud-based learning.

Overall, the results validate the feasibility of a low-cost, speech-driven autonomous wheelchair capable of reliable operation in real indoor environments. By explicitly analyzing performance limits and addressing current limitations through larger-scale user studies and enhanced perception strategies, the proposed platform has strong potential for future clinical and domestic applications.

## Conclusion and future work

In this paper, we presented the design, implementation, and evaluation of a voice-controlled smart wheelchair that integrates three key technological pillars: a deep learning–based speech recognition module, an autonomous navigation system built upon the Robot Operating System (ROS), and a mapping/localization framework relying on Simultaneous Localization and Mapping (SLAM). By combining these elements into a unified and modular architecture, our system demonstrates that intuitive human–machine interaction and robust autonomous mobility can be achieved simultaneously in an affordable, compact, and fully reproducible platform.

The experimental results confirmed the effectiveness, robustness, and real-time capabilities of the proposed solution. The adapted speech recognition engine achieved recognition rates above 90% in quiet conditions and maintained competitive performance under realistic noise levels, including TV audio, kitchen appliances, and crowded corridors. Navigation accuracy remained consistently below 10 cm in both simulation and real-world tests, while the end-to-end system latency averaged 0.8 s, ensuring responsive and comfortable interaction. The integration of a quantitatively defined safety layer—featuring dynamic speed regulation, emergency stop behavior, and multi-scan validation to avoid false triggers—further enhanced reliability, addressing an aspect often underdeveloped in comparable prototypes.

Beyond these quantitative results, the principal contribution of this work lies in its coherent end-to-end integration of components that are frequently studied in isolation. Our approach closes the gap between theoretical research and practical deployment by:

- Adapting and fine-tuning a lightweight ASR model to a restricted, domain-specific vocabulary—including impaired-speech samples—to improve accessibility for users with severe motor or articulation limitations.
- Leveraging ROS as a robust coordination framework linking speech input, SLAM-based localization, path planning, and safety supervision into a scalable and maintainable architecture.
- Implementing and validating a real-time SLAM pipeline suitable for low-cost embedded hardware, enabling reliable navigation without prior maps.
- Conducting a dual validation campaign in simulation (Gazebo/RViz) and in real-world indoor environments, thus moving beyond studies restricted to controlled or purely virtual conditions.

Beyond the functional integration of existing open-source components, the scientific contribution of this work lies in the methodological innovations required to deploy reliable autonomous navigation in assistive contexts. These include the introduction of a temporal-consistency filtering mechanism that improves SLAM robustness in dynamic indoor environments, the design of a real-time coordination layer linking SLAM, probabilistic localization, and voice-driven intent, and the development of a reproducible ground-truth evaluation protocol enabling quantitative assessment of mapping and localization accuracy. Such elements extend the system beyond a mere assembly of software modules and constitute methodological advances that address long-standing challenges identified in recent smart-wheelchair research.

From a broader perspective, this research contributes to the growing field of assistive robotics by providing a low-cost, reproducible, and user-centered platform that directly addresses key gaps identified in recent literature: limited robustness of speech interaction, insufficient transparency in safety mechanisms, and a general lack of real-world, system-level evaluation in smart wheelchair research.

Despite these promising results, several research directions remain open. First, the dependence on a single 2D LiDAR, although cost-effective, limits perception in highly dynamic or cluttered environments. Future work will explore multisensor fusion—combining LiDAR with RGB-D cameras, radar, or inertial units—to enhance robustness, especially in narrow corridors, reflective surfaces, or occlusions. Second, expanding the interaction modality toward multimodal and adaptive interfaces (voice, gaze, gestures, EEG/BCI) will improve accessibility for users with diverse functional profiles. Third, further progress may arise from energy-aware embedded AI models and reinforcement learning strategies that optimize autonomy and computational load.

A particularly critical direction for future research concerns large-scale user evaluations. As highlighted by multiple reviewers, comprehensive studies involving participants with different motor and speech impairments are necessary to assess usability, cognitive workload, trust, and long-term acceptance. Such evaluations require formal ethical approval and collaboration with clinical partners, and constitute the next major step before real-world deployment. Likewise, a more rigorous safety framework—including quantitative false positive/negative analysis, formal hazard assessment (e.g., HARA/FMEA), and consideration of international assistive mobility standards (ISO 13482, ISO 7176, IEC 60601-1)—will be central to future clinical translation.

Finally, integrating the wheelchair into a connected ecosystem of assistive technologies, enabling cloud-based adaptation, remote monitoring, and shared learning across devices, opens the door to large-scale deployment in rehabilitation centers and home environments.

In conclusion, the proposed system represents a significant step toward next-generation intelligent mobility-assistance solutions. By successfully integrating robust perception, safe navigation, and accessible voice interaction into a functional and low-cost prototype, this work provides both a rigorous research contribution and a realistic foundation for future clinical and domestic applications. Ultimately, it aims to contribute to greater autonomy, social inclusion, and quality of life for individuals with reduced mobility.

## Data Availability

The datasets generated and/or analysed during the current study, including simulation environments, mapping data and navigation experiments, are available from the corresponding author upon reasonable request. All simulation setups were created using the Gazebo and RViz frameworks and are reproducible following the description in the Methods section.

## Abbreviations

AMCL: Adaptive Monte Carlo localization
ASR: Automatic speech recognition
CPU: Central Processing Unit
DWA: Dynamic window approach
FPS: Frames per second
GPU: Graphics Processing Unit
GUI: Graphical user interface
IMU: Inertial measurement unit
LiDAR: Light detection and ranging
PID: Proportional–integral–derivative
RAM: Random Access Memory
ROS: Robot operating system
RViz: ROS visualization environment
SLAM: Simultaneous localization and mapping
WER: Word error rate
YOLO: You only look once

## Code availability

The code developed during this study is available from the corresponding author upon reasonable request.
